# Genome-wide mapping of miRNAs expressed in embryonic stem cells and pluripotent stem cells generated by different reprogramming strategies

**DOI:** 10.1186/1471-2164-15-488

**Published:** 2014-06-18

**Authors:** Botao Zhao, Dehua Yang, Jing Jiang, Jinsong Li, Chunsun Fan, Menggui Huang, Yi Fan, Yan Jin, Youxin Jin

**Affiliations:** School of Life Sciences, Shanghai University, Shanghai, 200444 China; The National Center for Drug Screening and the CAS Key Laboratory of Receptor Research, Shanghai Institute of Materia Medica, Chinese Academy of Sciences (CAS), Shanghai, 201203 China; State Key Laboratory of Cell Biology, Institute of Biochemistry and Cell Biology, Institutes for Biological Sciences, Chinese Academy of Sciences, Shanghai, 200031 China; Department of etiology, Qidong liver cancer institute, Qidong, Jiangsu 226200 China; Department of radiation oncology, University of Pennsylvania, Philadelphia, PA 19104 USA; Institute of Biomedicine and Biotechnology, Shenzhen Institutes of Advanced Technology, Chinese Academy of Sciences, Shenzhen, 518055 China

## Abstract

**Background:**

Reprogrammed cells, including induced pluripotent stem cells (iPSCs) and nuclear transfer embryonic stem cells (NT-ESCs), are similar in many respects to natural embryonic stem cells (ESCs). However, previous studies have demonstrated that iPSCs retain a gene expression signature that is unique from that of ESCs, including differences in microRNA (miRNA) expression, while NT-ESCs are more faithfully reprogrammed cells and have better developmental potential compared with iPSCs.

**Results:**

We focused on miRNA expression and explored the difference between ESCs and reprogrammed cells, especially ESCs and NT-ESCs. We also compared the distinct expression patterns among iPSCs, NT-ESCs and NT-iPSCs. The results demonstrated that reprogrammed cells (iPSCs and NT-ESCs) have unique miRNA expression patterns compared with ESCs. The comparison of differently reprogrammed cells (NT-ESCs, NT-iPSCs and iPSCs) suggests that several miRNAs have key roles in the distinct developmental potential of reprogrammed cells.

**Conclusions:**

Our data suggest that miRNAs play a part in the difference between ESCs and reprogrammed cells, as well as between MEFs and pluripotent cells. The variation of miRNA expression in reprogrammed cells derived using different reprogramming strategies suggests different characteristics induced by nuclear transfer and iPSC generation, as well as different developmental potential among NT-ESCs, iPSCs and NT-iPSCs.

**Electronic supplementary material:**

The online version of this article (doi:10.1186/1471-2164-15-488) contains supplementary material, which is available to authorized users.

## Background

Embryonic stem cell (ESC) research has made remarkable progress since the establishment of the first human embryonic stem cell line in 1998 [1]. The pluripotent nature of ESCs makes them valuable as a tool to model embryonic development and for regenerative medicine *in vitro*. They are also valuable as a cell resource for transplantation. However, the ethical issues surrounding the derivation of ESCs from embryos hinders the clinical application of ESCs and many countries limit or ban their use [2].

In 2006, Yamanaka brought pluripotent cell research into a new era by showing that over-expression of four key transcription factors, Oct4, Sox2, Klf4 and c-Myc, could reprogram mouse somatic cells into ESC-like cells that showed similar morphology and pluripotent nature to that of ESCs [3]. They named these ESCs-like cells “induced pluripotent stem cells” (iPSCs). Research into iPSCs has since proceeded at an astonishing pace and has included the establishment of human iPSCs and high efficiency induction of iPSCs with fewer transcription factors in combination with microRNAs (miRNAs) or small compounds [4–9]. With ongoing advances in miRNA biology, these findings may lead to a nonviral, nontranscription-factor mediated procedure for generating iPSCs for use not only in basic stem cell biology studies, but also in high throughput generation of human iPSC clones from large patient populations.

Despite the robustness of iPSCs technology, human somatic cell nuclear transfer (SCNT) research remains an important approach for regenerative medicine [10, 11]. The recent establishment of human pluripotent ESCs by SCNT has been long-anticipated as an approach for generating patient-matched nuclear transfer (NT)-ESCs for studies of disease mechanisms and for developing specific therapies [12]. Since the initial discovery in amphibians in 1962, SCNT success in a range of different mammalian species has demonstrated that such reprogramming activity is universal [12–14]. Direct comparisons between iPSCs and NT-ESCs in the mouse indicated that SCNT-based reprogramming is more efficient in resetting the epigenetic identity of parental somatic cells [15, 16]. The breakthrough discovery of such a reprogramming event provides a powerful means to generate and regenerate unlimited pluripotent stem cells directly from body tissue cells. Yet, full understanding of the mechanism involved, called somatic cell reprogramming (SCR), remains elusive.

iPSCs share the majority properties with ESCs, such as morphology, differentiation, pluripotency, DNA methylation and gene expression; however, there is a wide range of evidence showing that there are subtle yet substantial differences between these cell types [17–22]. These studies demonstrated that iPSCs are characterized by a unique gene and miRNA expression signature as well as a CpG methylation pattern that distinguishes them from ESCs. Recently, several miRNAs have been shown to enhance iPSC reprogramming when expressed with combinations of the four key factors [23, 24]. These miRNAs belong to families of miRNAs that are expressed preferentially in ESCs and are thought to help maintain the ESC phenotype. How these miRNAs enhance iPSC reprogramming is unclear but may involve their ability to regulate the cell cycle. Further experiments demonstrated that the miR-302/367 cluster can directly reprogram mouse and human somatic cells to an iPSCs state in the absence of any of the previously described iPSCs transcription factors [25–29]. These results show that miRNAs may be the crucial factors of iPSCs as well as having key roles in their induction.

The purpose of this study was to determine the miRNA profiles and to identify the differentially expressed miRNAs in ESCs, reprogrammed cells and mouse embryonic fibroblasts (MEFs) by deep sequencing analysis. Previous studies reported that reprogrammed cells generated by different reprogramming strategies showed different developmental potential [16]. We therefore generated three kinds of mouse reprogrammed cells: iPSCs, NT-ESCs and NT-iPSCs. We evaluated the differences between the miRNAs signatures of ESCs, MEFs and the variously derived reprogrammed cells.

## Results

### Sequencing the small RNA transcriptomes in different cell lines

**Figure 1:**
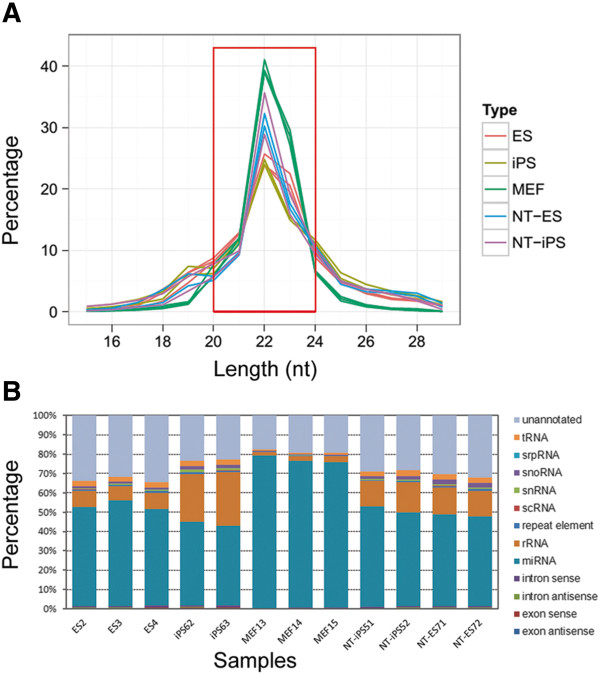
**Deep sequencing of the small RNA transcriptomes in MEF, ESCs and reprogrammed cells. A**. Read length distribution (nt) after removal of adaptors, low quality tags and contaminants. The y-axis depicts the percentage of read lengths relative to the total number of reads in each dataset. Red box outlines the peak of read length ranging from 20 to 24 nt. **B**. Proportion of total/unique small tags matched to different RNA species. The sequences obtained from each sample were subjected to a series of sequence similarity searches using specific databases (listed at right).

To generate miRNA profiles of ESCs, reprogrammed cells and MEFs by deep sequencing analysis, we used three different batches of RNA samples for MEFs and ESCs, and two different batches of RNA samples for NT-ESCs, iPSCs and NT-iPSCs (which were all derived from the same MEF line). Small RNA transcriptomes from 12 RNA samples from five different cell lines were analyzed by next generation Solexa sequencing. The total sequencing reads are shown in Additional file 1: Table S1, which shows that high quality sequence was generated based on the high percent of clean reads and the high consistency among the different batches of RNA samples from the same cell lines. After removal of adapter sequence, low quality tags and contaminants, analysis of all reads showed the read length distribution main peaks at 20–24 nt. This indicates that mature miRNAs were enriched in the sequencing samples (Figure 1A). The composition of small RNAs depicted in Figure 1B illustrates the different distribution of RNA species in the different cell lines. The MEF cells expressed more miRNAs than ESCs or the reprogrammed cells.

### Distinct miRNA expression signatures are associated with differently derived cells

**Figure 2:**
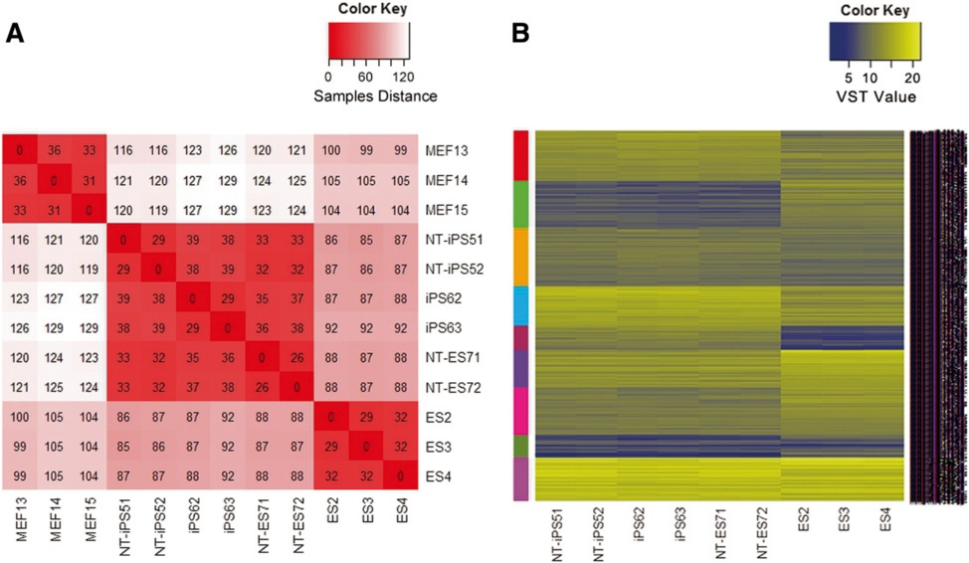
**Distinct expression pattern of miRNAs between ESCs and iPSCs. A**. A heatmap showing the distances between the samples as calculated from the variance stabilizing transformed data. The distance of two samples is indicated in the crossing box of the two samples. A larger number means less similarity between two samples. **B**. miRNAs (VST > 10) were grouped by k-means clustering based on the different expression patterns between ESCs and iPSCs. Each group of miRNAs is indicated by a colored sidebar.

Previous studies have reported different miRNA expression profiles in ESCs and iPSCs [17]. In addition, reprogrammed cells generated by different reprogramming strategies show different developmental potential [16]. However, detailed miRNAs profiles of these cells have not so far been explored. We, therefore, evaluated differences among miRNA signatures of differently derived cells. As shown in Additional file 2: Figure S1, clustering analyses of all samples based on miRNA expression demonstrated a significant difference between MEFs and pluripotent cells, as well as major similarities among the differently derived pluripotent stem cells. The heatmap representing the distances between the samples also showed the same signature (Figure 2A). However, despite similarities in the pluripotent nature and the miRNA expression profiles of ESCs and reprogrammed cells, a significant difference in the miRNA expression pattern was still evident after clustering analysis, which is indicated by a colored sidebar in Figure 2B. In addition, the three reprogrammed cell types also presented obvious differences in miRNAs expression (Figure 2B).

### Differentially expressed miRNAs in ESCs and reprogrammed cells

**Figure 3:**
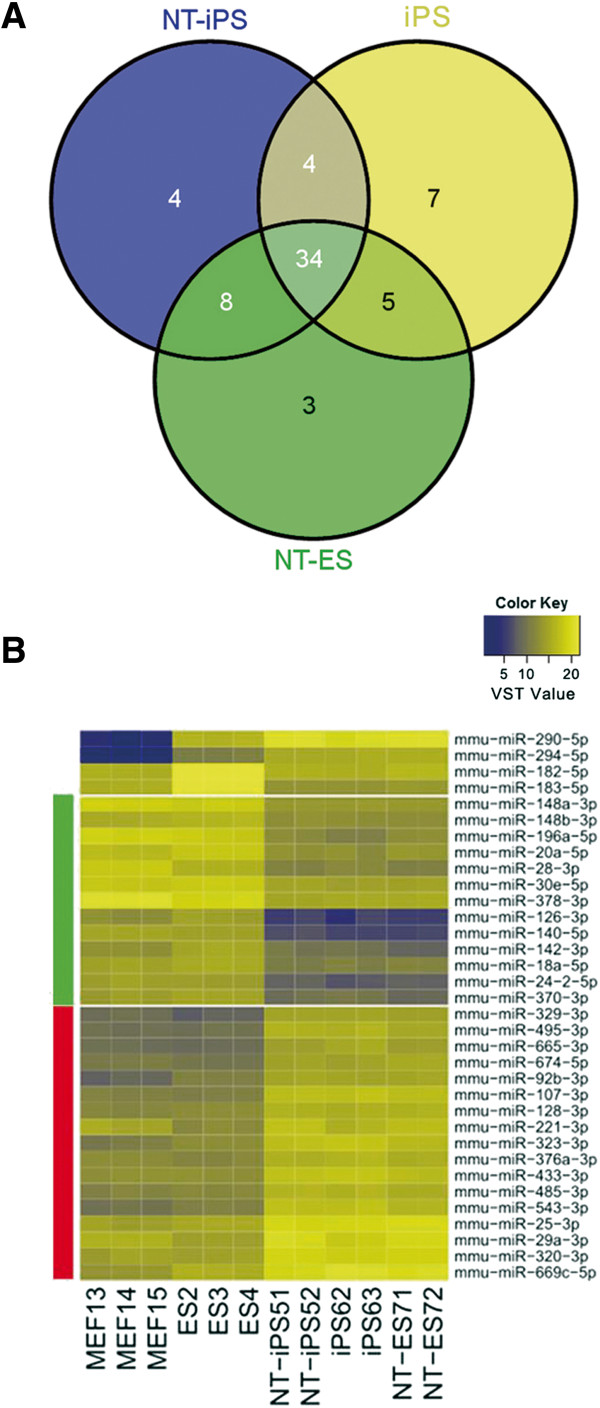
**Differentially expressed miRNAs in ESCs and reprogrammed cells. A**. Venn Diagram shows the number of differentially expressed miRNAs in ESCs and the three reprogrammed cell types. The top 50 miRNAs that are differentially expressed between ESCs and each reprogrammed cell type are represented in each circle. The intersection of the three circles indicates 34 miRNAs that are commonly differentially expressed between ESCs and reprogrammed cells. **B**. Heatmap shows the 34 common reprogrammed cell-specific miRNAs. Except for miR-294, 295, 182, 183, mentioned above, the expression of the remaining miRNAs was similar in ESCs and MEFs. These miRNAs were grouped into two major classes indicated by green and red sidebars. The expression level of the green class of miRNAs was lower in reprogrammed cells than in ESCs and MEFs and the expression level of the red class was higher in reprogrammed cells than in ESCs and MEFs.

**Figure 4:**
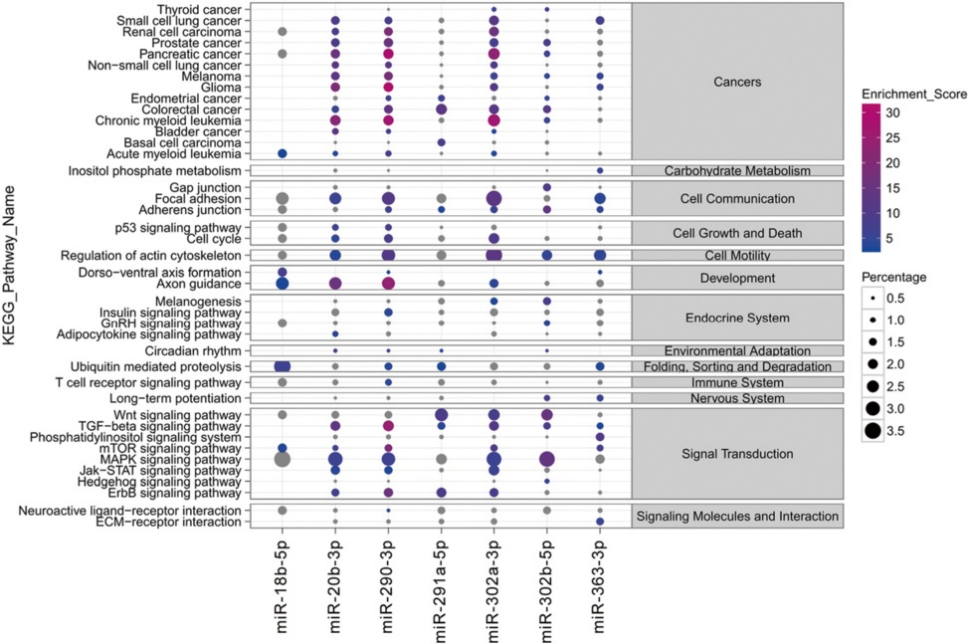
**Target gene prediction.** Target genes of miRNAs in the four pluripotency related classes were predicted by microT v4.0 and mapped to KEGG pathways by mirPath. Not all miRNAs in the four pluripotency-related classes were analyzed because some of them are not yet included in the mirPath database. Some miRNAs that have less than 100 target genes were also excluded in this plot. The bubble plot shows the KEGG pathway enrichment of some pluripotency related miRNAs. The bubble color scaled the enrichment score. A larger score means more significant enrichment. The size of the bubble scaled the percentage of the enriched target genes among total target miRNAs of a miRNA. KEGG pathway names are listed at the left of the plot and the function class names of the pathways are listed in the right panel.

To determine whether miRNAs are expressed in ESCs and reprogrammed pluripotent cells at similar levels, expression profiling of all known miRNAs was performed on mouse ESCs and reprogrammed pluripotent cells, including iPSCs, NT-ESCs and NT-iPSCs. A Venn diagram shows the top 50 miRNAs that were differentially expressed between each reprogrammed cell type and ESCs, and indicates 34 miRNAs that were differentially expressed in all reprogrammed pluripotent cells, compared to mouse ESCs (Figure 3A, Additional file 3: Table S2). The detailed expression profiles of the 34 miRNAs are illustrated in a heatmap (Figure 3B). Among them, the expression of miR-290, miR-294, miR-182 and miR-183 were lower in MEF cells than in pluripotent cells. However, the expression of the other 30 miRNAs was similar in ESCs and MEFs but was significantly different in reprogrammed cells compared to ESCs or MEFs (Figure 3B). In detail, 13 miRNAs were highly expressed and 17 miRNAs were expressed at low levels in reprogrammed cells and were significantly different compared to those in ESCs. The miRNAs showing the maximum difference between MEFs and pluripotent cells were miR-290 and miR-294, while miR-126 and miR-140 showed the maximum difference between reprogrammed cells and somatic cells. These results indicate that the difference between ESCs and reprogrammed cells might be attributed, at least in part, to differently expressed miRNAs. The following target prediction analysis using bioinformatics described the KEGG pathway and the result showed the enrichment of target genes diffused distribution which was different from the pluripotent specific miRNAs (Additional file 4: Figure S2, Additional file 5: Table S3, and Figure 4).

### Distinct expression of miRNAs in the three types of reprogrammed cell

**Figure 5:**
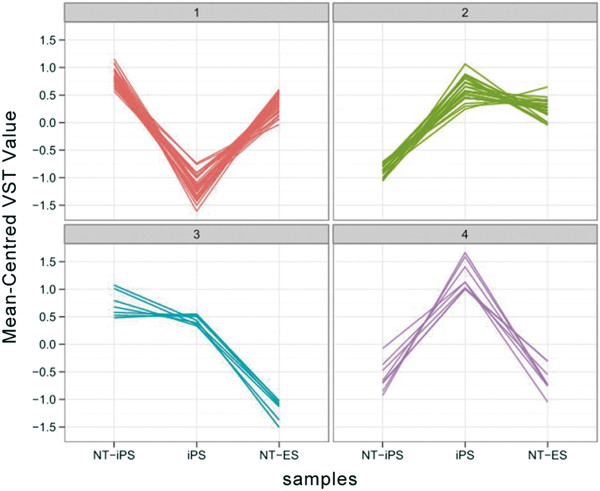
**Top differentially expressed miRNAs in three kinds of reprogrammed cells.** The differentially expressed miRNAs were grouped by k-means clustering. MiRNAs with VST values of more than 10 in at least one reprogrammed cells with an adjusted p value less than 0.05 (ANOVA) were included in this analysis. For profiling, miRNA expression value was centred to the mean.

In addition to studying differences in miRNA expression profiles between ESCs and reprogrammed cells, we also compared the expression of miRNAs in the differently derived reprogrammed cells, NT-ESCs, iPCSc and NT-iPSCs. Our previous study demonstrated that different reprogramming strategies resulted in distinct developmental potential and found that NT-ESCs had better developmental potential compared with iPSCs. Furthermore, subsequent nuclear transfer to iPSCs did not rescue, but rather increased the developmental deficiency of iPSCs, resulting in NT-iPSCs having reduced developmental ability [16]. Our current miRNA analysis produced four clusters that were differentially expressed among the different pluripotent cells in which groups 1, 2 and 4 represented differences that were common to iPSCs and the nuclear transfer strategy, while group 3 represented the sequential decreasing expression similar as the developmental ability in different pluripotent cells (Figure 5, Additional file 6: Table S4). The critical miRNAs were expressed differently between differently reprogrammed cells, suggesting that iPSCs had a miRNA signature that defines them as unique from ESCs and nuclear transfer-derived cells.

### MiRNAs potentially contribute to the pluripotency of ESCs and reprogrammed cells

**Figure 6:**
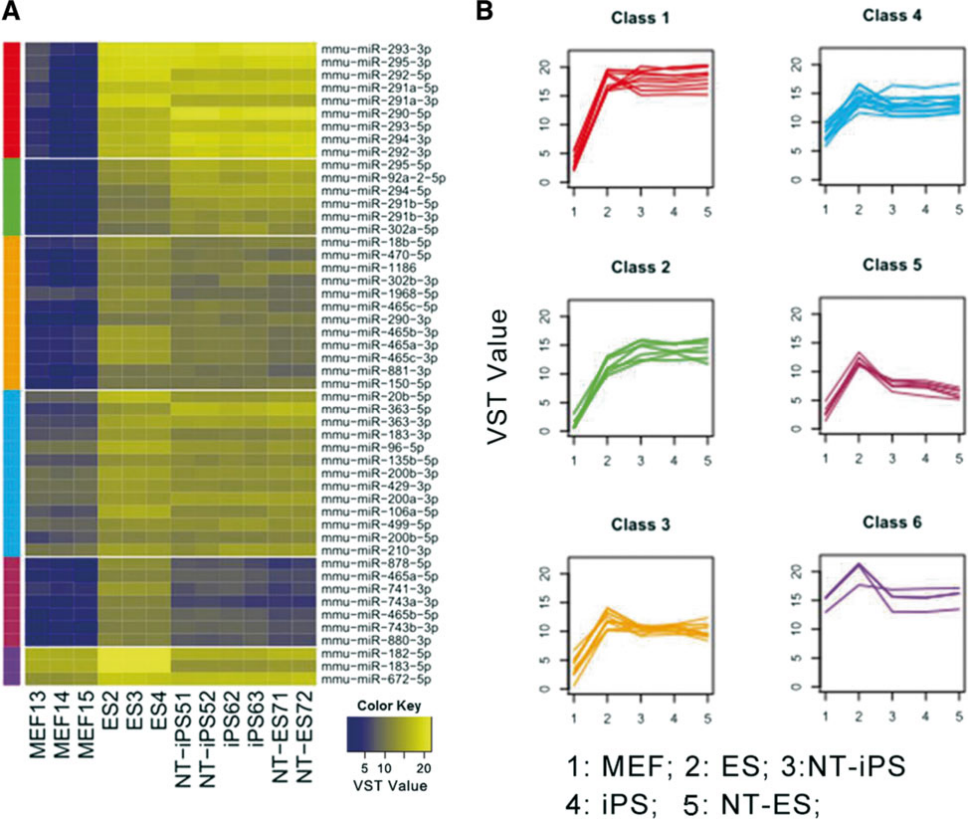
**MiRNAs potentially contribute to the pluripotency of ESCs and iPSCs. A**. The top 50 differentially expressed miRNAs in ESCs and MEF cells were grouped as six classes by k-means clustering. Each miRNA class is indicated by colored sidebars. The first four classes of miRNAs were considered as pluripotency-related miRNAs. VST counts value is scaled by a color key. **B**. The expression profiles across all samples of the first four classes of miRNAs are plotted. The color of each plot was in accordance with the color of the sidebar in Figure 3A.

**Figure 7:**
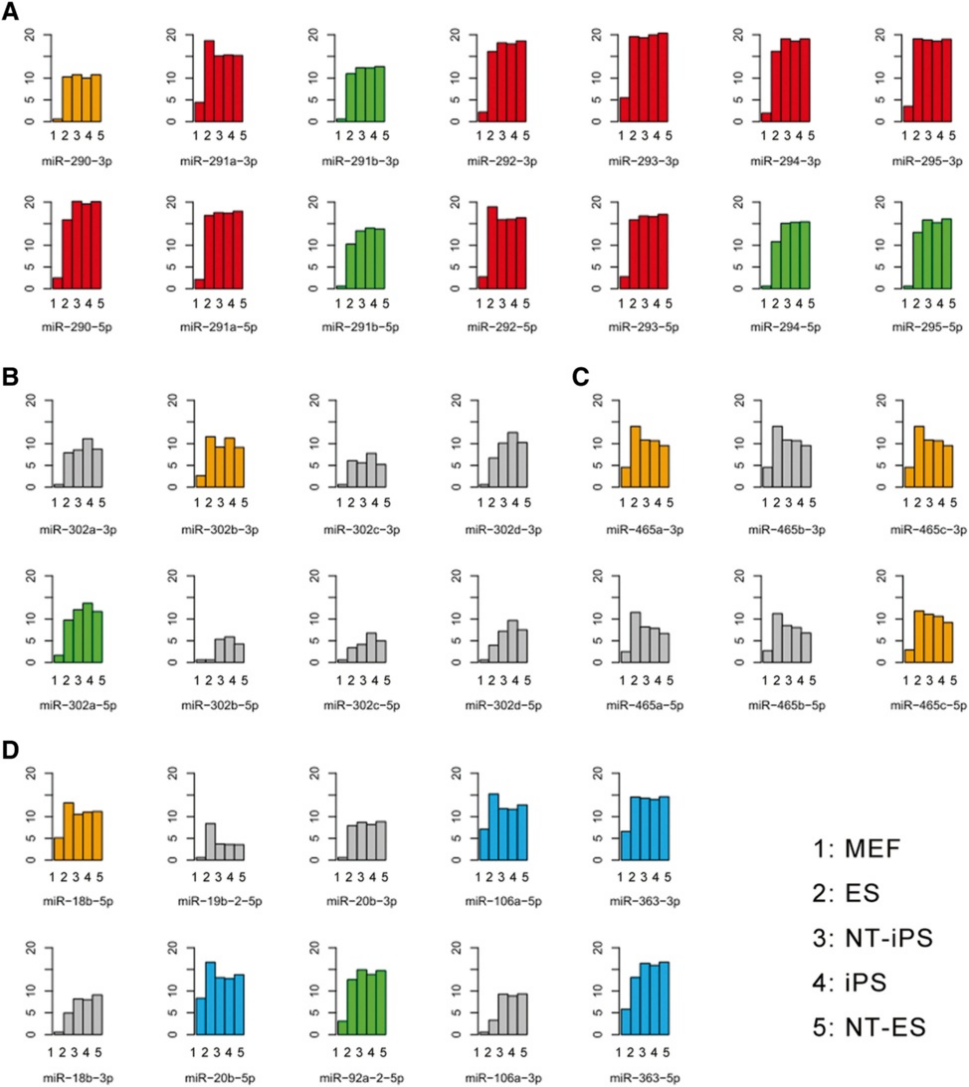
**Histograms of the miRNAs from the four major pluripotency related miRNA clusters.** Histograms illustrate the expression of the miRNAs from the four major miRNA clusters identified in the four classes of pluripotency-related miRNAs (Additional file 9: Table S7). **A**. miR-290-295 cluster. **B**. miR-302a-302d cluster. **C**. miR-465a-465c cluster. **D**. miR-18b-363 cluster. The color of each plot is in accordance with the color of the sidebar in Figure 3A. The gray plots indicate the miRNAs that were not called by the criteria of the top 50 differentially expressed miRNAs but they are located in the listed miRNAs clusters.

A significant difference was present between the miRNA profiles of MEFs and pluripotent cells. Figure 6A and Additional file 7: Table S5 list the top 50 differentially expressed miRNAs which were grouped into six classes by k-means clustering (Figure 6B, Additional file 8: Table S6). Interestingly, all 50 miRNAs were expressed at significantly higher levels in pluripotent cells than in MEF cells which demonstrated that these miRNAs were pluripotent specific. Figure 7 and Additional file 9: Table S7 illustrate the four major families of pluripotent-specific miRNAs, including miR-290, miR-302 and miR-465 families, which are widely recognized to be pluripotent specific. The miRNA families clustered together on pluripotent cells, as well as the data from our sequencing analysis, demonstrated the crucial roles of these miRNAs in pluripotency. Ensemble data shows the analysis of the four clustering miRNAs, indicating that upstream sequence elements of these miRNAs were mainly ESCs-specific transcript factor binding sites, DNase 1 footprint protection sites, polymerase protection sites or histone modification features (Additional file 10: Figure S3), which further demonstrated the pluripotent signature of these miRNAs. We then analyzed the target genes of the miRNAs in the four pluripotency related classes by microT v4.0 and mapped these genes to KEGG pathways using mirPath. The target genes were mostly enriched in cancer related and signal transduction pathways, which are also characteristic of immortalized cells, such as pluripotent cells (Figure 4, Additional file 11: Table S8).

## Discussion

We prepared 12 samples from five different cell lines to analyze miRNA expression profiles. The consistent miRNA expression profiles generated from different batches of samples from the same cell lines indicated accurate sample preparation and sequencing. Meanwhile, high quality sequences with a high percentage of clean reads were achieved in all samples. Moreover, our data from different pluripotent cells, including iPSCs, NT-ESCs, ESCs and NT-iPSCs present similar miRNAs features as compared with MEF miRNAs.

Previous studies have demonstrated the distinct signatures of iPSCs and ESCs for gene expression, epigenetic and miRNAs profiles [17]. In the present study, we examined three kinds of reprogrammed cells to identify differences in miRNA expression between reprogrammed cells and ESCs. Thirty-four miRNAs were identified to be differentially expressed in the three reprogrammed cells compared with ESCs, among which miR-24 and miR-370 were previously reported in literature. From the heatmap depiction of miRNA expression, we found that miRNAs were differentially expressed not only relative to ESCs but also to MEFs, which showed similar expression levels compared with reprogrammed cells, suggesting that reprogrammed cells have miRNAs signatures different to those of somatic cells. The target gene prediction and mapping to KEGG pathways illustrated the diffused distribution of target genes of differentially expressed miRNAs for both highly and lowly expressed miRNAs in reprogrammed cells.

To detect differences between ESCs and reprogrammed cells, we compared miRNA expression in different reprogrammed cells, derived using different strategies, including iPSCs, NT-ESCs and NT-iPSCs all of which were derived from the same MEFs. Our previous data suggested different development potential with NT-ESCs > iPSCs > NT-iPSCs. In the present study, we identified differentially expressed miRNAs in the three kinds of reprogrammed cells. The k-means clustering analysis showed four miRNA groups with significant variations. Group 1 represented the variation of NT-ESCs and NT-iPSCs > iPSCs, group 2 and 4 showed the difference of iPSCs > NT-ESCs and NT-iPSCs, which demonstrated that the nuclear transfer strategy induced different expression of miRNAs compared with the strategy of using four transcription factors to induce iPSCs. Meanwhile, group 3 with NT-ESCs > iPSCs > NT-iPSCs represented variation that was similar to previously demonstrated sequential development potential. Previously, studies have reported that miR-302 could replace all transcription factors to reprogram somatic cells to iPSCs [27–30]. Interestingly, the miR-302 family was represented in group 4 and was highly expressed in iPSCs, indicating that iPSCs might be more highly dependent on miR-302 expression than pluripotent cells produced by the SCNT method.

It has been clearly shown that various types of cell vary not only in the expression of their coding genes, but also in the expression of their noncoding genes. In the present study, we compared differences in miRNAs expression between MEFs and ESCs and among MEF-derived iPSCs, NT-ESCs and NT-iPSCs to identify pluripotent specific miRNAs. The 50 top differentially expressed miRNAs were assigned to four clusters which were almost all highly expressed in pluripotent cells. Among them, miR-290 and miR-302 clusters were identified in previous studies to play key roles in pluripotency maintenance. The consistency of our results with those in the literature demonstrate the reliability of our sequence data. Furthermore, the target gene analysis showed that four miRNAs clusters mainly targeted genes involved in cancers and signal transduction pathways. The common characteristics of cell proliferation and immortalization are shared between cancer cells and pluripotent cells, as are activated signal transduction pathways.

## Conclusions

In conclusion, we first report differentially expressed miRNAs among ESCs, MEFs and three kinds of reprogrammed cells. The unique expression of miRNAs in pluripotent cells mainly represents acquired expression of miRNAs, while the higher and lower expression levels of miRNAs in ESCs compared with reprogrammed cells may reflect the difference between naturally pluripotent cells and reprogrammed cells. Finally, the variation in miRNA expression among reprogrammed cells derived using different reprogramming strategies suggests different characteristics induced by nuclear transfer and iPSC generation, as well as different developmental potential among NT-ESCs, iPSCs and NT-iPSCs.

## Methods

### Cell culture

Mouse ES cell line (E14) was maintained in our lab. iPSCs were obtained by infecting MEFs (C57B6/129SvJae F1) with a dox-inducible lentivirus carrying the four reprogramming factors (Oct4, Sox2, Klf4 and c-Myc). NT-ESCs were established by reprogramming MEFs into ESCs using nuclear transfer. To establish NT-iPSCs, the nucleus of an iPSC was transferred into an enucleated oocyte. NT-iPSCs were established to reflect the combination of nuclear transfer and iPS technologies. iPSCs, NT-ESCs, and NT-iPSCs were derived from the same MEF cells. All the cells were cultured and maintained as described previously [16]. All experiments were approved by the Ethics Committee of Shanghai Institute of Biochemistry and Cell Biology.

### RNA preparation and sequencing

Total RNA was isolated using TRIzol reagent. RNA quality was assessed with an Agilent 2100 bioanalyzer. Total RNAs from MEFs, ESCs and the three reprogrammed cell types were subjected to Solexa sequencing, performed by BGI-Shenzhen, Shenzhen, China. The sequencing data have been deposited with the Gene Expression Omnibus repository (http://www.ncbi.nlm.nih.gov/geo) under accession number GSE52950.

### Data analysis and statistics

After removal of adaptors, low quality tags and contaminants from the sequenced tags, clean reads were annotated. MiRNA reads were analyzed using the DESeq package [31] in R language [32]. Normalization and variance stabilizing transformations (VST) were performed before further analysis. Differently expressed miRNAs and sample distances between any two kinds of samples were calculated by DESeq. MiRNAs with variance stabilizing transformed values of more than 10 were clustered using the gplots package [33].

We present a hypothesis that miRNAs contributing to pluripotency should meet at least two criteria. First, these miRNAs should be highly expressed in ESCs and expressed at lower levels in MEF cells. Second, these miRNAs should also show relatively high levels of expression in iPSCs. Based on these criteria, the expression profiles of the top 50 miRNAs that were more highly expressed in ESCs than in MEF cells were grouped by k-means clustering using the Vegan package [34]. The genome context of each miRNA was extracted from miRbase [35–38] and presented using the Ensembl Genome Browser. Target genes of miRNAs were predicted and enriched in KEGG pathways using mirPath [39]. The enrichment results are presented in a “bubble plot” using the ggplot2 package [40].

To identify iPSC-specific miRNAs, the top 50 miRNAs differently expressed between ESCs and each reprogrammed cell type were screened and 34 miRNAs that were commonly differentially expressed between ESCs and all reprogrammed cell types were identified as iPSC-specific miRNAs. These miRNAs were grouped by k-means clustering. The target genes of these miRNAs were also mapped to KEGG pathways.

To identify differentially expressed miRNAs in the three reprogrammed cell types, miRNAs with a VST value more than 10 in at least one reprogrammed cell type were analyzed. MiRNAs with an adjusted p value less than 0.05 (ANOVA) were identified as differentially expressed miRNAs in these different reprogrammed cells, and were grouped by k-means clustering using the Vegan package.

## Electronic supplementary material

Additional file 1: Table S1: Description of sequencing data. (DOCX 17 KB)

Additional file 2: Figure S1: Distinct expression patterns of miRNAs between MEFs and pluripotent cells. Clustering analysis of all samples based on miRNA expression whose counts was more than 10 after variance stabilizing transformation in at least one sample (VST > 10). (JPEG 1 MB)

Additional file 3: Table S2: 50 most differentially expressed miRNAs in iPSCs and ESCs. (XLSX 46 KB)

Additional file 4: Figure S2: The KEGG pathway enrichment of the target genes of the two classes of miRNA. Not all miRNAs in the four pluripotency related classes were analyzed because some of them are not yet included in the database. Some miRNAs that have less than 100 target genes were also excluded in this plot. The bubble plot shows the KEGG pathway enrichment of some pluripotency related miRNAs. The bubble color scaled the enrichment score. A larger score means more significant enrichment. The size of the bubble scaled the percentage of the enriched target genes among total target miRNAs of a miRNA. KEGG pathway names are listed at the left of the plot and the function class names of the pathways are listed in the right panel. (JPEG 2 MB)

Additional file 5: Table S3: KEGG pathway analysis of target genes that showed the most difference among the three reprogramming cells and ESCs. MiRNAs in the “gain” group were highly expressed in the three reprogrammed cells but lowly expressed in ESCs. MiRNAs in the “loss” group were highly expressed in ESCs but lowly expressed in the three reprogrammed cells. (XLS 68 KB)

Additional file 6: Table S4: Differently expressed miRNAs (VST value more than 10 and adjusted p value less than 0.05) were grouped by k-means clustering. Four groups were identified. “n” means these miRNA didn’t fall in any groups. (XLSX 17 KB)

Additional file 7: Table S5: Top 50 differentially expressed miRNAs in ESCs and MEF cells. (DOCX 29 KB)

Additional file 8: Table S6: Six classes of miRNA grouped by k-means from the 50 differentially expressed miRNAs in ESCs and MEF cells. (DOCX 19 KB)

Additional file 9: Table S7: MiRNA gene clusters identified in the first four classes of pluripotency-related miRNAs. ‘nc’ means that these miRNAs are not in any classes. (DOCX 19 KB)

Additional file 10: Figure S3: Ensemble gene browser image showing the four miRNA clusters identified in the four classes of pluripotency-related miRNAs. ESC-specific transcript factor binding sites, DNase 1 footprint protection sites, polymerase protection sites and histone modification features are indicated. (JPEG 2 MB)

Additional file 11: Table S8: miRNA target genes enriched in KEGG pathways. ‘Counts’ means the number of target genes that mapped to the corresponding pathway. (DOCX 38 KB)

## Abbreviations

iPSCs: induced pluripotent stem cells
NT-ESCs: Nuclear transfer embryonic stem cells
ESCs: Embryonic stem cells
miRNA: microRNA
SCNT: Somatic cell nuclear transfer
NT: Nuclear transfer
SCR: Somatic cell reprogramming
MEF: Mouse embryonic fibroblast
VST: Variance stabilizing transformations.
