# Supplementary material for: Genome-wide mapping of miRNAs expressed in embryonic stem cells and pluripotent stem cells generated by different reprogramming strategies
Source: BMC Genomics. 2014 Jun 18;15(1):488. doi: 10.1186/1471-2164-15-488 (PMC4082626; doi:10.1186/1471-2164-15-488)
Supplement: Supplementary file 1 — Additional file 1: Table S1: Description of sequencing data. (DOCX 17 KB) [file 12864_2014_6194_MOESM1_ESM.docx]

Table S1 Description of sequencing data.

| type | ES2 | | ES3 | | ES4 | | MEF13 | | MEF14 | | MEF15 | |
| --- | --- | --- | --- | --- | --- | --- | --- | --- | --- | --- | --- | --- |
|  | counts | % | counts | % | counts | % | counts | % | counts | % | counts | % |
| total reads | 10813833 |  | 11420205 |  | 11588740 |  | 11374007 |  | 10966715 |  | 11213722 |  |
| high quality | 10631726 | 100% | 11245168 | 100% | 11358769 | 100% | 11186447 | 100% | 10744938 | 100% | 10996666 | 100% |
| adaptor3 null | 172955 | 1.63% | 83917 | 0.75% | 86138 | 0.76% | 33905 | 0.30% | 116551 | 1.08% | 30305 | 0.28% |
| insert null | 369925 | 3.48% | 216672 | 1.93% | 380877 | 3.35% | 116348 | 1.04% | 193778 | 1.80% | 138273 | 1.26% |
| adaptor5 contaminants | 157172 | 1.48% | 104741 | 0.93% | 182867 | 1.61% | 30822 | 0.28% | 34402 | 0.32% | 32634 | 0.30% |
| smaller than 18nt | 502490 | 4.73% | 370661 | 3.30% | 681292 | 6.00% | 184177 | 1.65% | 126900 | 1.18% | 177449 | 1.61% |
| polyA | 1188 | 0.01% | 1013 | 0.01% | 1255 | 0.01% | 41 | 0.00% | 166 | 0.00% | 144 | 0.00% |
| **clean reads** | **9427996** | **88.68%** | **10468164** | **93.09%** | **10026340** | **88.27%** | **10821154** | **96.73%** | **10273141** | **95.61%** | **10617861** | **96.56%** |

Table 1 (continued)

| type | NT-iPS51 | | NT-iPS52 | | iPS62 | | iPS63 | | NT-ES71 | | NT-ES72 | |
| --- | --- | --- | --- | --- | --- | --- | --- | --- | --- | --- | --- | --- |
|  | count | % | count | % | count | % | count | % | count | % | count | % |
| total reads | 14670602 |  | 15815722 |  | 13073136 |  | 15481918 |  | 16401271 |  | 14119784 |  |
| high quality | 14499956 | 100% | 15657288 | 100% | 12889947 | 100% | 15268338 | 100% | 16181357 | 100% | 13930879 | 100% |
| adaptor3 null | 8480 | 0.06% | 6765 | 0.04% | 14152 | 0.11% | 16466 | 0.11% | 9109 | 0.06% | 6233 | 0.04% |
| insert null | 25220 | 0.17% | 36901 | 0.24% | 38984 | 0.30% | 50751 | 0.33% | 24227 | 0.15% | 27211 | 0.20% |
| adaptor5 contaminants | 343390 | 2.37% | 445945 | 2.85% | 327649 | 2.54% | 386995 | 2.53% | 496274 | 3.07% | 476417 | 3.42% |
| smaller than 18nt | 194289 | 1.34% | 709888 | 4.53% | 572762 | 4.44% | 425782 | 2.79% | 262377 | 1.62% | 374331 | 2.69% |
| polyA | 2844 | 0.02% | 3585 | 0.02% | 536 | 0.00% | 1168 | 0.01% | 8290 | 0.05% | 3056 | 0.02% |
| **clean reads** | **13925733** | **96.04%** | **14454204** | **92.32%** | **11935864** | **92.60%** | **14387176** | **94.23%** | **15381080** | **95.05%** | **13043631** | **93.63%** |
