# Supplementary figures and images for: Genome-wide mapping of miRNAs expressed in embryonic stem cells and pluripotent stem cells generated by different reprogramming strategies
Source: BMC Genomics. 2014 Jun 18;15(1):488. doi: 10.1186/1471-2164-15-488 (PMC4082626; doi:10.1186/1471-2164-15-488)

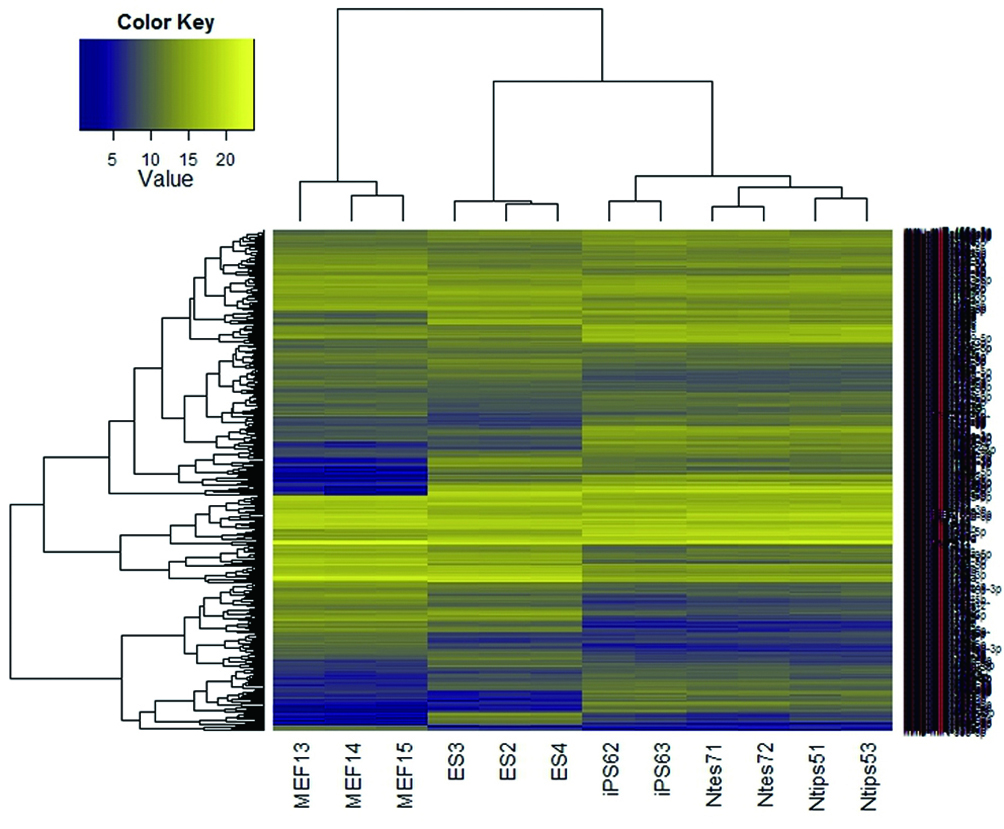

Supplement: Supplementary file 2 — Additional file 2: Figure S1: Distinct expression patterns of miRNAs between MEFs and pluripotent cells. Clustering analysis of all samples based on miRNA expression whose counts was more than 10 after variance stabilizing transformation in at least one sample (VST > 10). (JPEG 1 MB) [file 12864_2014_6194_MOESM2_ESM.jpeg]

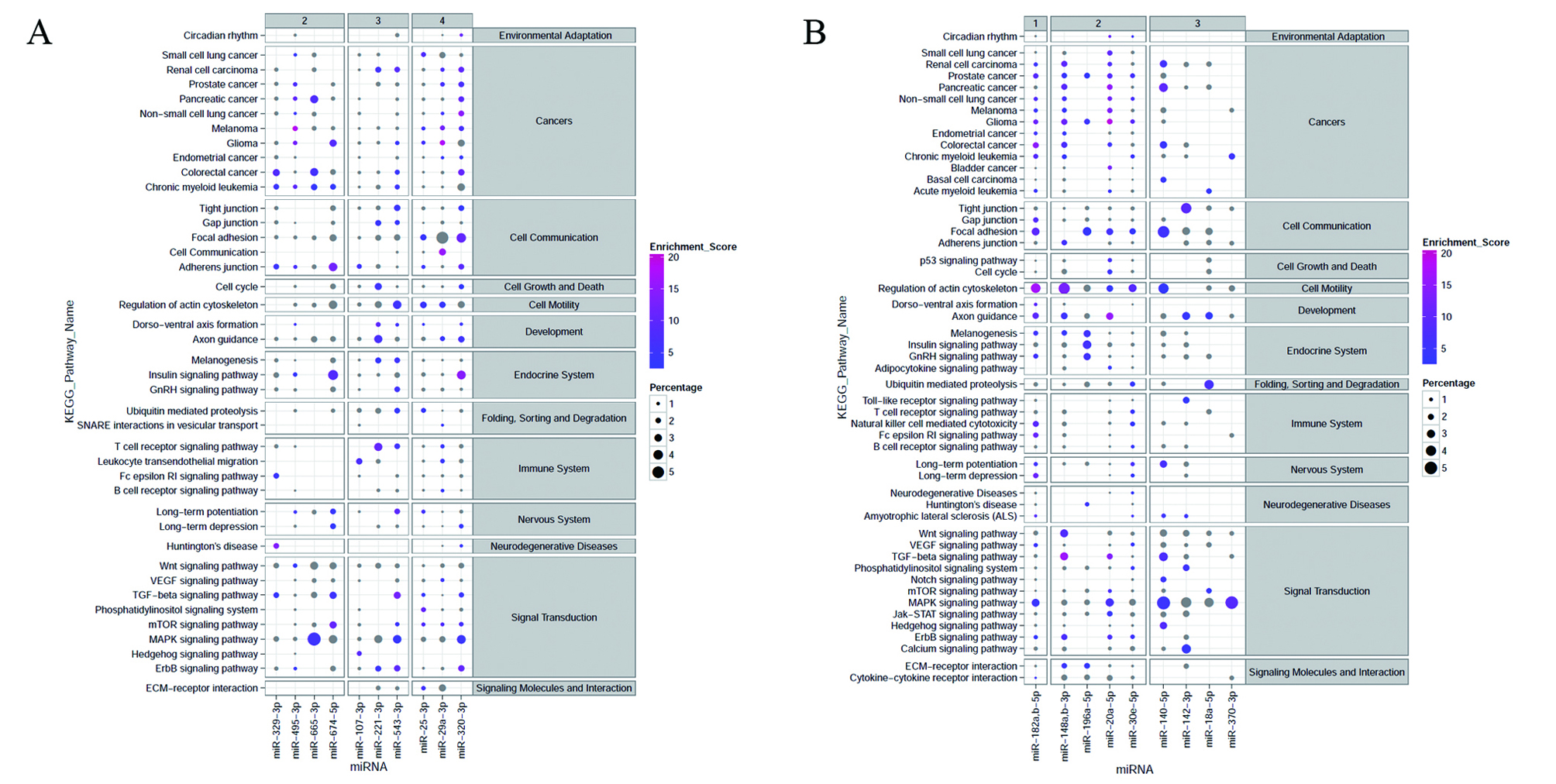

Supplement: Supplementary file 4 — Additional file 4: Figure S2: The KEGG pathway enrichment of the target genes of the two classes of miRNA. Not all miRNAs in the four pluripotency related classes were analyzed because some of them are not yet included in the database. Some miRNAs that have less than 100 target genes were also excluded in this plot. The bubble plot shows the KEGG pathway enrichment of some pluripotency related miRNAs. The bubble color scaled the enrichment score. A larger score means more significant enrichment. The size of the bubble scaled the percentage of the enriched target genes among total target miRNAs of a miRNA. KEGG pathway names are listed at the left of the plot and the function class names of the pathways are listed in the right panel. (JPEG 2 MB) [file 12864_2014_6194_MOESM4_ESM.jpeg]

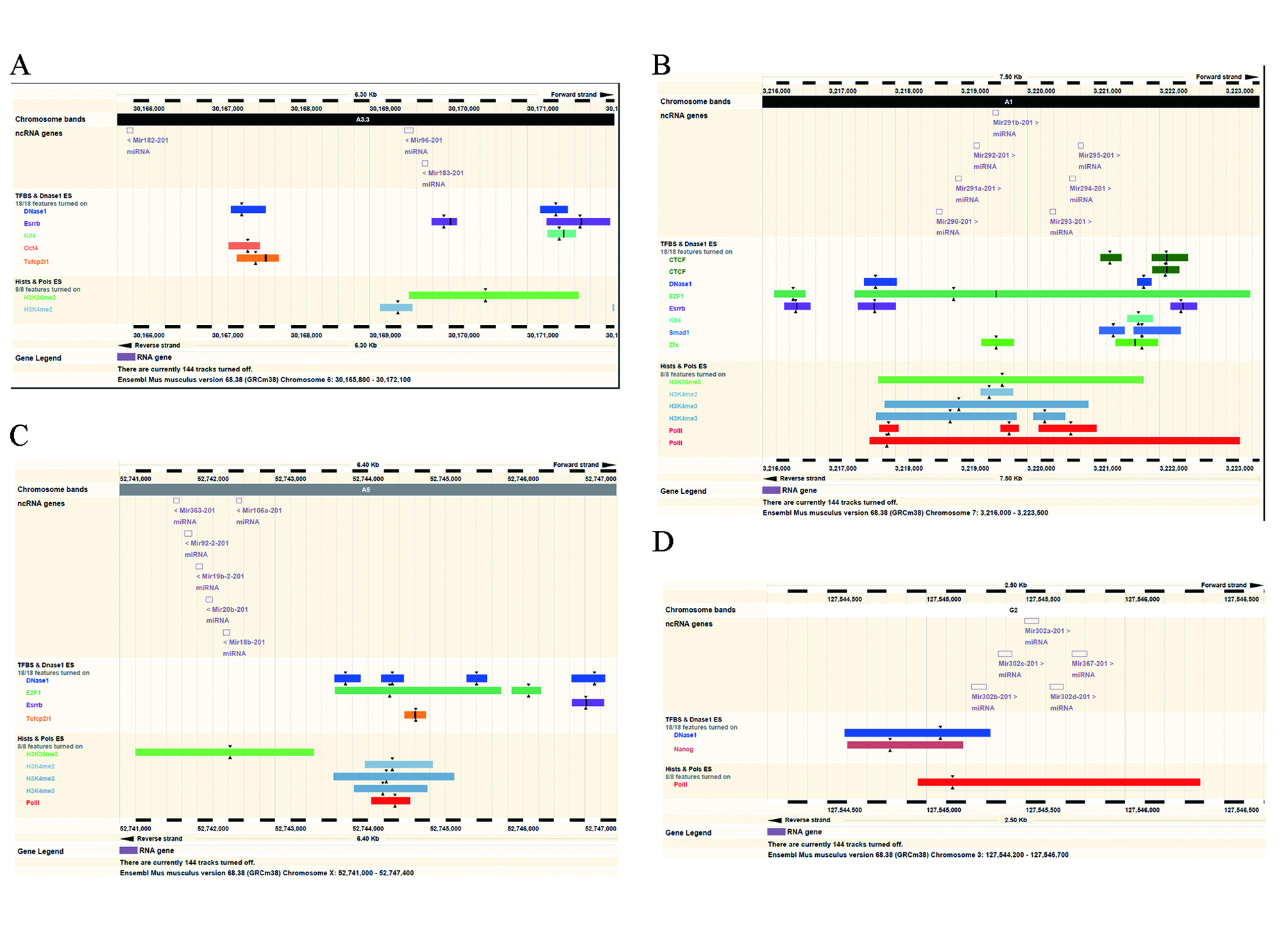

Supplement: Supplementary file 10 — Additional file 10: Figure S3: Ensemble gene browser image showing the four miRNA clusters identified in the four classes of pluripotency-related miRNAs. ESC-specific transcript factor binding sites, DNase 1 footprint protection sites, polymerase protection sites and histone modification features are indicated. (JPEG 2 MB) [file 12864_2014_6194_MOESM10_ESM.jpeg]
