# Supplementary material for: Genome-wide mapping of miRNAs expressed in embryonic stem cells and pluripotent stem cells generated by different reprogramming strategies
Source: BMC Genomics. 2014 Jun 18;15(1):488. doi: 10.1186/1471-2164-15-488 (PMC4082626; doi:10.1186/1471-2164-15-488)
Supplement: Supplementary file 7 — Additional file 7: Table S5: Top 50 differentially expressed miRNAs in ESCs and MEF cells. (DOCX 29 KB) [file 12864_2014_6194_MOESM7_ESM.docx]

Table S5. The top 50 differentially expressed miRNAs in ESCs and MEF cells.

|  | MEF13 | MEF14 | MEF15 | ES2 | ES3 | ES4 | NT-iPS51 | NT-iPS52 | iPS62 | iPS63 | NT-ES71 | NT-ES72 | class |
| --- | --- | --- | --- | --- | --- | --- | --- | --- | --- | --- | --- | --- | --- |
| mmu-miR-293-3p | 7.23 | 4.87 | 4.26 | 19.50 | 19.57 | 19.62 | 19.58 | 19.02 | 19.73 | 20.23 | 20.36 | 20.29 | 1 |
| mmu-miR-295-3p | 7.00 | 3.00 | 0.59 | 18.76 | 19.24 | 19.14 | 18.96 | 18.62 | 18.24 | 18.82 | 18.96 | 18.93 | 1 |
| mmu-miR-292-5p | 7.06 | 0.59 | 0.59 | 18.84 | 18.84 | 19.06 | 15.75 | 16.18 | 16.06 | 15.99 | 16.33 | 16.41 | 1 |
| mmu-miR-291a-5p | 5.21 | 0.59 | 0.59 | 16.93 | 16.73 | 16.94 | 17.45 | 17.69 | 17.32 | 17.48 | 17.80 | 17.96 | 1 |
| mmu-miR-291a-3p | 6.36 | 3.00 | 3.78 | 18.45 | 18.59 | 18.85 | 15.30 | 14.97 | 15.29 | 15.27 | 15.36 | 15.06 | 1 |
| mmu-miR-290-5p | 3.82 | 0.59 | 3.03 | 15.81 | 15.80 | 16.13 | 20.14 | 20.23 | 19.22 | 19.96 | 19.99 | 20.21 | 1 |
| mmu-miR-293-5p | 4.68 | 0.59 | 3.03 | 15.68 | 15.93 | 16.16 | 16.84 | 16.76 | 16.74 | 16.50 | 17.05 | 17.20 | 1 |
| mmu-miR-294-3p | 4.68 | 0.59 | 0.59 | 16.16 | 15.96 | 16.43 | 19.26 | 18.76 | 18.19 | 18.89 | 19.11 | 18.92 | 1 |
| mmu-miR-292-3p | 5.41 | 0.59 | 0.59 | 16.05 | 16.13 | 16.14 | 17.85 | 18.41 | 17.59 | 18.17 | 18.35 | 18.81 | 1 |
| mmu-miR-295-5p | 0.59 | 0.59 | 0.59 | 12.78 | 12.94 | 13.31 | 15.82 | 15.92 | 15.06 | 15.40 | 16.07 | 16.08 | 2 |
| mmu-miR-92a-2-5p | 3.07 | 3.00 | 3.03 | 12.45 | 12.71 | 12.76 | 14.89 | 14.97 | 13.74 | 13.97 | 14.98 | 14.45 | 2 |
| mmu-miR-294-5p | 0.59 | 0.59 | 0.59 | 10.79 | 10.75 | 11.04 | 15.04 | 15.13 | 15.05 | 15.53 | 15.44 | 15.46 | 2 |
| mmu-miR-291b-5p | 0.59 | 0.59 | 0.59 | 10.31 | 10.22 | 10.38 | 13.19 | 13.49 | 14.07 | 13.83 | 13.83 | 13.74 | 2 |
| mmu-miR-291b-3p | 0.59 | 0.59 | 0.59 | 10.83 | 11.06 | 11.26 | 12.71 | 12.17 | 12.77 | 11.97 | 12.74 | 12.64 | 2 |
| mmu-miR-302a-5p | 0.59 | 0.59 | 3.78 | 9.64 | 9.88 | 9.83 | 12.45 | 11.86 | 13.52 | 13.79 | 11.90 | 11.62 | 2 |
| mmu-miR-18b-5p | 4.97 | 4.87 | 5.54 | 13.01 | 13.44 | 13.22 | 10.63 | 10.44 | 10.77 | 11.32 | 11.23 | 11.23 | 3 |
| mmu-miR-470-5p | 3.07 | 0.59 | 3.78 | 12.00 | 12.10 | 11.71 | 10.16 | 10.59 | 10.64 | 9.83 | 8.85 | 9.32 | 3 |
| mmu-miR-1186 | 4.31 | 0.59 | 3.03 | 11.68 | 11.74 | 11.73 | 10.74 | 10.88 | 10.75 | 11.68 | 12.25 | 12.44 | 3 |
| mmu-miR-302b-3p | 4.31 | 0.59 | 3.03 | 11.66 | 11.62 | 11.62 | 9.53 | 8.94 | 11.29 | 11.31 | 9.28 | 9.01 | 3 |
| mmu-miR-1968-5p | 7.00 | 6.96 | 6.31 | 12.43 | 12.79 | 12.68 | 9.05 | 9.38 | 9.53 | 9.48 | 9.31 | 9.39 | 3 |
| mmu-miR-465c-5p | 4.31 | 3.74 | 0.59 | 12.09 | 11.91 | 11.55 | 10.76 | 11.48 | 10.92 | 10.40 | 9.11 | 9.28 | 3 |
| mmu-miR-290-3p | 0.59 | 0.59 | 0.59 | 10.30 | 10.31 | 10.35 | 10.60 | 10.98 | 9.91 | 10.17 | 10.82 | 10.78 | 3 |
| mmu-miR-465b-3p | 4.31 | 4.22 | 5.15 | 14.31 | 13.96 | 13.77 | 10.65 | 11.08 | 10.92 | 10.49 | 9.39 | 9.79 | 3 |
| mmu-miR-465a-3p | 4.31 | 4.22 | 5.15 | 14.31 | 13.96 | 13.77 | 10.65 | 11.08 | 10.92 | 10.49 | 9.39 | 9.79 | 3 |
| mmu-miR-465c-3p | 4.31 | 4.22 | 5.15 | 14.31 | 13.96 | 13.76 | 10.64 | 11.08 | 10.92 | 10.49 | 9.39 | 9.78 | 3 |
| mmu-miR-881-3p | 4.31 | 0.59 | 3.03 | 10.29 | 10.09 | 10.25 | 10.19 | 9.68 | 10.04 | 9.79 | 8.29 | 8.47 | 3 |
| mmu-miR-150-5p | 3.07 | 3.00 | 4.62 | 10.23 | 10.31 | 10.20 | 9.97 | 10.22 | 9.87 | 10.24 | 10.78 | 10.66 | 3 |
| mmu-miR-20b-5p | 8.24 | 8.37 | 8.39 | 16.53 | 16.81 | 16.57 | 13.26 | 12.95 | 12.97 | 12.77 | 13.88 | 13.74 | 4 |
| mmu-miR-363-5p | 5.59 | 5.65 | 6.20 | 12.86 | 13.39 | 13.19 | 16.46 | 16.37 | 15.77 | 16.16 | 16.83 | 16.55 | 4 |
| mmu-miR-363-3p | 6.79 | 6.53 | 6.49 | 14.32 | 14.53 | 14.81 | 14.16 | 14.41 | 14.15 | 13.80 | 14.60 | 14.67 | 4 |
| mmu-miR-183-3p | 7.06 | 7.28 | 7.55 | 13.47 | 13.31 | 13.33 | 11.04 | 11.11 | 11.22 | 11.11 | 11.60 | 11.84 | 4 |
| mmu-miR-96-5p | 9.84 | 10.16 | 9.93 | 15.51 | 15.61 | 15.59 | 13.15 | 13.27 | 13.11 | 12.46 | 13.30 | 13.18 | 4 |
| mmu-miR-135b-5p | 7.34 | 7.23 | 7.23 | 12.48 | 12.84 | 12.81 | 12.13 | 11.55 | 12.09 | 11.82 | 12.19 | 11.84 | 4 |
| mmu-miR-200b-3p | 9.67 | 8.87 | 9.46 | 14.43 | 14.26 | 14.20 | 12.50 | 12.96 | 13.03 | 12.72 | 13.20 | 13.65 | 4 |
| mmu-miR-429-3p | 9.39 | 9.37 | 9.45 | 13.98 | 13.89 | 13.47 | 12.29 | 12.34 | 12.03 | 12.35 | 12.15 | 12.48 | 4 |
| mmu-miR-200a-3p | 9.05 | 8.99 | 9.25 | 13.61 | 13.52 | 13.27 | 14.17 | 13.93 | 14.21 | 14.52 | 14.32 | 14.30 | 4 |
| mmu-miR-106a-5p | 7.53 | 6.69 | 7.23 | 14.97 | 15.58 | 15.20 | 11.87 | 11.85 | 11.80 | 11.66 | 12.95 | 12.55 | 4 |
| mmu-miR-499-5p | 8.95 | 8.37 | 8.32 | 12.55 | 12.92 | 13.00 | 12.60 | 12.42 | 13.67 | 13.55 | 12.52 | 13.17 | 4 |
| mmu-miR-200b-5p | 6.15 | 7.18 | 7.71 | 11.78 | 11.41 | 11.72 | 10.73 | 11.27 | 11.06 | 11.01 | 11.46 | 11.79 | 4 |
| mmu-miR-210-3p | 10.09 | 9.74 | 10.23 | 14.18 | 13.97 | 13.77 | 12.25 | 13.04 | 14.38 | 14.32 | 13.91 | 14.27 | 4 |
| mmu-miR-878-5p | 4.31 | 3.00 | 0.59 | 12.51 | 12.41 | 12.38 | 7.57 | 7.36 | 7.87 | 6.61 | 5.16 | 5.90 | 5 |
| mmu-miR-465a-5p | 3.07 | 3.74 | 0.59 | 11.56 | 11.52 | 11.54 | 8.28 | 8.12 | 8.17 | 7.54 | 6.71 | 6.66 | 5 |
| mmu-miR-741-3p | 5.41 | 4.58 | 4.62 | 13.51 | 13.50 | 13.14 | 7.02 | 7.76 | 7.95 | 7.09 | 5.67 | 6.43 | 5 |
| mmu-miR-743a-3p | 3.82 | 3.00 | 3.78 | 11.54 | 11.51 | 11.60 | 6.40 | 6.58 | 6.12 | 5.35 | 5.28 | 5.05 | 5 |
| mmu-miR-465b-5p | 0.59 | 3.74 | 3.78 | 11.27 | 11.37 | 11.16 | 8.31 | 8.74 | 8.14 | 7.96 | 6.58 | 7.00 | 5 |
| mmu-miR-743b-3p | 0.59 | 3.00 | 0.59 | 10.83 | 11.03 | 11.08 | 8.85 | 8.36 | 8.71 | 8.23 | 7.20 | 7.34 | 5 |
| mmu-miR-880-3p | 0.59 | 3.00 | 3.78 | 11.70 | 11.16 | 11.41 | 7.57 | 7.36 | 7.66 | 8.15 | 6.71 | 6.81 | 5 |
| mmu-miR-182-5p | 15.64 | 15.44 | 15.35 | 21.33 | 21.43 | 21.42 | 15.63 | 15.64 | 15.39 | 15.55 | 16.25 | 16.23 | 6 |
| mmu-miR-183-5p | 15.21 | 15.40 | 15.46 | 21.08 | 21.03 | 21.13 | 13.05 | 13.02 | 13.07 | 12.91 | 13.57 | 13.37 | 6 |
| mmu-miR-672-5p | 12.88 | 12.71 | 13.21 | 17.83 | 17.84 | 17.37 | 16.79 | 16.93 | 17.15 | 16.93 | 17.00 | 17.27 | 6 |
