# Supplementary material for: Genome-wide mapping of miRNAs expressed in embryonic stem cells and pluripotent stem cells generated by different reprogramming strategies
Source: BMC Genomics. 2014 Jun 18;15(1):488. doi: 10.1186/1471-2164-15-488 (PMC4082626; doi:10.1186/1471-2164-15-488)
Supplement: Supplementary file 8 — Additional file 8: Table S6: Six classes of miRNA grouped by k-means from the 50 differentially expressed miRNAs in ESCs and MEF cells. (DOCX 19 KB) [file 12864_2014_6194_MOESM8_ESM.docx]

Table S6. The six classes of miRNA grouped by k-means from the 50 differentially expressed miRNAs in ESCs and MEF cells.

|  | MEF | ESCs | NT-iPSCs | iPSCs | NT-ESCs | class |
| --- | --- | --- | --- | --- | --- | --- |
| mmu-miR-293-3p | 5.45 | 19.56 | 19.30 | 19.98 | 20.32 | 1 |
| mmu-miR-295-3p | 3.53 | 19.05 | 18.79 | 18.53 | 18.95 | 1 |
| mmu-miR-292-5p | 2.74 | 18.92 | 15.96 | 16.03 | 16.37 | 1 |
| mmu-miR-291a-5p | 2.13 | 16.87 | 17.57 | 17.40 | 17.88 | 1 |
| mmu-miR-291a-3p | 4.38 | 18.63 | 15.14 | 15.28 | 15.21 | 1 |
| mmu-miR-290-5p | 2.48 | 15.92 | 20.18 | 19.59 | 20.10 | 1 |
| mmu-miR-293-5p | 2.76 | 15.92 | 16.80 | 16.62 | 17.12 | 1 |
| mmu-miR-294-3p | 1.95 | 16.18 | 19.01 | 18.54 | 19.02 | 1 |
| mmu-miR-292-3p | 2.19 | 16.11 | 18.13 | 17.88 | 18.58 | 1 |
| mmu-miR-295-5p | 0.59 | 13.01 | 15.87 | 15.23 | 16.07 | 2 |
| mmu-miR-92a-2-5p | 3.03 | 12.64 | 14.93 | 13.85 | 14.71 | 2 |
| mmu-miR-294-5p | 0.59 | 10.86 | 15.08 | 15.29 | 15.45 | 2 |
| mmu-miR-291b-5p | 0.59 | 10.31 | 13.34 | 13.95 | 13.79 | 2 |
| mmu-miR-291b-3p | 0.59 | 11.05 | 12.44 | 12.37 | 12.69 | 2 |
| mmu-miR-302a-5p | 1.65 | 9.78 | 12.15 | 13.65 | 11.76 | 2 |
| mmu-miR-18b-5p | 5.12 | 13.22 | 10.54 | 11.05 | 11.23 | 3 |
| mmu-miR-470-5p | 2.48 | 11.94 | 10.37 | 10.23 | 9.09 | 3 |
| mmu-miR-1186 | 2.64 | 11.72 | 10.81 | 11.22 | 12.34 | 3 |
| mmu-miR-302b-3p | 2.64 | 11.63 | 9.24 | 11.30 | 9.15 | 3 |
| mmu-miR-1968-5p | 6.75 | 12.63 | 9.21 | 9.50 | 9.35 | 3 |
| mmu-miR-465c-5p | 2.88 | 11.85 | 11.12 | 10.66 | 9.20 | 3 |
| mmu-miR-290-3p | 0.59 | 10.32 | 10.79 | 10.04 | 10.80 | 3 |
| mmu-miR-465b-3p | 4.56 | 14.01 | 10.87 | 10.70 | 9.59 | 3 |
| mmu-miR-465a-3p | 4.56 | 14.01 | 10.87 | 10.70 | 9.59 | 3 |
| mmu-miR-465c-3p | 4.56 | 14.01 | 10.86 | 10.70 | 9.59 | 3 |
| mmu-miR-881-3p | 2.64 | 10.21 | 9.94 | 9.91 | 8.38 | 3 |
| mmu-miR-150-5p | 3.56 | 10.24 | 10.10 | 10.06 | 10.72 | 3 |
| mmu-miR-20b-5p | 8.33 | 16.64 | 13.11 | 12.87 | 13.81 | 4 |
| mmu-miR-363-5p | 6.60 | 14.56 | 14.28 | 13.97 | 14.64 | 4 |
| mmu-miR-363-3p | 5.82 | 13.15 | 16.42 | 15.96 | 16.69 | 4 |
| mmu-miR-183-3p | 7.30 | 13.37 | 11.08 | 11.16 | 11.72 | 4 |
| mmu-miR-96-5p | 9.98 | 15.57 | 13.21 | 12.79 | 13.24 | 4 |
| mmu-miR-135b-5p | 7.27 | 12.71 | 11.84 | 11.95 | 12.01 | 4 |
| mmu-miR-200b-3p | 9.33 | 14.30 | 12.73 | 12.87 | 13.43 | 4 |
| mmu-miR-429-3p | 9.40 | 13.78 | 12.32 | 12.19 | 12.32 | 4 |
| mmu-miR-200a-3p | 9.10 | 13.47 | 14.05 | 14.36 | 14.31 | 4 |
| mmu-miR-106a-5p | 7.15 | 15.25 | 11.86 | 11.73 | 12.75 | 4 |
| mmu-miR-499-5p | 8.54 | 12.82 | 12.51 | 13.61 | 12.85 | 4 |
| mmu-miR-200b-5p | 7.01 | 11.64 | 11.00 | 11.04 | 11.62 | 4 |
| mmu-miR-210-3p | 10.02 | 13.97 | 12.64 | 14.35 | 14.09 | 4 |
| mmu-miR-878-5p | 2.63 | 12.43 | 7.46 | 7.24 | 5.53 | 5 |
| mmu-miR-465a-5p | 2.46 | 11.54 | 8.20 | 7.86 | 6.69 | 5 |
| mmu-miR-741-3p | 4.87 | 13.38 | 7.39 | 7.52 | 6.05 | 5 |
| mmu-miR-743a-3p | 3.53 | 11.55 | 6.49 | 5.74 | 5.16 | 5 |
| mmu-miR-465b-5p | 2.70 | 11.27 | 8.53 | 8.05 | 6.79 | 5 |
| mmu-miR-743b-3p | 1.39 | 10.98 | 8.61 | 8.47 | 7.27 | 5 |
| mmu-miR-880-3p | 2.45 | 11.42 | 7.46 | 7.91 | 6.76 | 5 |
| mmu-miR-182-5p | 15.47 | 21.39 | 15.63 | 15.47 | 16.24 | 6 |
| mmu-miR-183-5p | 15.35 | 21.08 | 13.03 | 12.99 | 13.47 | 6 |
| mmu-miR-672-5p | 12.93 | 17.68 | 16.86 | 17.04 | 17.13 | 6 |
