# Supplementary material for: Genome-wide mapping of miRNAs expressed in embryonic stem cells and pluripotent stem cells generated by different reprogramming strategies
Source: BMC Genomics. 2014 Jun 18;15(1):488. doi: 10.1186/1471-2164-15-488 (PMC4082626; doi:10.1186/1471-2164-15-488)
Supplement: Supplementary file 9 — Additional file 9: Table S7: MiRNA gene clusters identified in the first four classes of pluripotency-related miRNAs. ‘nc’ means that these miRNAs are not in any classes. (DOCX 19 KB) [file 12864_2014_6194_MOESM9_ESM.docx]

Table S7. MiRNA gene clusters identified in the first four classes of pluripotency related miRNAs. ‘nc’ means that these miRNAs are not in any classes.

| mature miRNA | MEF | ES | NT-iPS | iPS | NT-ES | class | miRNA hairpin | Chromosome | Start | End | Strand |
| --- | --- | --- | --- | --- | --- | --- | --- | --- | --- | --- | --- |
| miR-290-3p | 0.59 | 10.32 | 10.79 | 10.04 | 10.80 | 3 | miR-290 | 7 | 3218627 | 3218709 | + |
| miR-290-5p | 2.48 | 15.92 | 20.18 | 19.59 | 20.10 | 1 |  |  |  |  |  |
| miR-291a-3p | 4.38 | 18.63 | 15.14 | 15.28 | 15.21 | 1 | miR-291a | 7 | 3218920 | 3219001 | + |
| miR-291a-5p | 2.13 | 16.87 | 17.57 | 17.40 | 17.88 | 1 |  |  |  |  |  |
| miR-291b-3p | 0.59 | 11.05 | 12.44 | 12.37 | 12.69 | 2 | miR-291b | 7 | 3219483 | 3219561 | + |
| miR-291b-5p | 0.59 | 10.31 | 13.34 | 13.95 | 13.79 | 2 |  |  |  |  |  |
| miR-292-3p | 2.19 | 16.11 | 18.13 | 17.88 | 18.58 | 1 | miR-292 | 7 | 3219190 | 3219271 | + |
| miR-292-5p | 2.74 | 18.92 | 15.96 | 16.03 | 16.37 | 1 |  |  |  |  |  |
| miR-293-3p | 5.45 | 19.56 | 19.30 | 19.98 | 20.32 | 1 | miR-293 | 7 | 3220344 | 3220423 | + |
| miR-293-5p | 2.76 | 15.92 | 16.80 | 16.62 | 17.12 | 1 |  |  |  |  |  |
| miR-294-3p | 1.95 | 16.18 | 19.01 | 18.54 | 19.02 | 1 | miR-294 | 7 | 3220642 | 3220725 | + |
| miR-294-5p | 0.59 | 10.86 | 15.08 | 15.29 | 15.45 | 2 |  |  |  |  |  |
| miR-295-3p | 3.53 | 19.05 | 18.79 | 18.53 | 18.95 | 1 | miR-295 | 7 | 3220774 | 3220842 | + |
| miR-295-5p | 0.59 | 13.01 | 15.87 | 15.23 | 16.07 | 2 |  |  |  |  |  |
| miR-302a-3p | 0.59 | 7.90 | 8.57 | 11.17 | 8.77 | nc | miR-302a | 3 | 1.27E+08 | 1.27E+08 | + |
| miR-302a-5p | 1.65 | 9.78 | 12.15 | 13.65 | 11.76 | 2 |  |  |  |  |  |
| miR-302b-3p | 2.64 | 11.63 | 9.24 | 11.30 | 9.15 | 3 | miR-302b | 3 | 1.27E+08 | 1.27E+08 | + |
| miR-302b-5p | 0.59 | 0.59 | 5.35 | 5.90 | 4.27 | nc |  |  |  |  |  |
| miR-302c-3p | 0.59 | 6.10 | 5.60 | 7.78 | 5.21 | nc | miR-302c | 3 | 1.27E+08 | 1.27E+08 | + |
| miR-302c-5p | 0.59 | 3.38 | 4.13 | 6.76 | 4.98 | nc |  |  |  |  |  |
| miR-302d-3p | 0.59 | 6.71 | 10.16 | 12.60 | 10.28 | nc | miR-302d | 3 | 1.27E+08 | 1.27E+08 | + |
| miR-302d-5p | 0.59 | 3.96 | 7.22 | 9.69 | 7.52 | nc |  |  |  |  |  |
| miR-465a-3p | 4.56 | 14.01 | 10.87 | 10.70 | 9.59 | 3 | miR-465a | X | 64092227 | 64092300 | - |
| miR-465a-5p | 2.46 | 11.54 | 8.20 | 7.86 | 6.69 | nc |  |  |  |  |  |
| miR-465b-3p | 4.56 | 14.01 | 10.87 | 10.70 | 9.59 | nc | miR-465b-1 | X | 64082377 | 64082455 | - |
| miR-465b-5p | 2.70 | 11.27 | 8.53 | 8.05 | 6.79 | nc | miR-465b-2 | X | 64088939 | 64089017 | - |
| miR-465c-3p | 4.56 | 14.01 | 10.86 | 10.70 | 9.59 | 3 | miR-465c-1 | X | 64079130 | 64079210 | - |
| miR-465c-5p | 2.88 | 11.85 | 11.12 | 10.66 | 9.20 | 3 | miR-465c-2 | X | 64085692 | 64085772 | - |
| miR-18b-5p | 5.12 | 13.22 | 10.54 | 11.05 | 11.23 | 3 | miR-18b | X | 50095508 | 50095590 | - |
| miR-18b-3p | 0.59 | 4.93 | 8.17 | 8.00 | 9.12 | nc |  |  |  |  |  |
| miR-19b-2-5p | 0.59 | 8.39 | 3.70 | 3.57 | 3.53 | nc | miR-19b-2 | X | 50095160 | 50095243 | - |
| miR-20b-5p | 8.33 | 16.64 | 13.11 | 12.87 | 13.81 | 4 | miR-20b | X | 50095290 | 50095369 | - |
| miR-20b-3p | 0.59 | 7.95 | 8.69 | 8.19 | 8.86 | nc |  |  |  |  |  |
| miR-92a-2-5p | 3.03 | 12.64 | 14.93 | 13.85 | 14.71 | 2 | miR-92a-2 | X | 50095015 | 50095105 | - |
| miR-106a-5p | 7.15 | 15.25 | 11.86 | 11.73 | 12.75 | 4 | miR-106a | X | 50095680 | 50095744 | - |
| miR-106a-3p | 0.59 | 3.27 | 9.30 | 8.88 | 9.35 | nc |  |  |  |  |  |
| miR-363-3p | 6.60 | 14.56 | 14.28 | 13.97 | 14.64 | 4 | miR-363 | X | 50094870 | 50094944 | - |
| miR-363-5p | 5.82 | 13.15 | 16.42 | 15.96 | 16.69 | 4 |  |  |  |  |  |
