# Supplementary material for: Genome-wide mapping of miRNAs expressed in embryonic stem cells and pluripotent stem cells generated by different reprogramming strategies
Source: BMC Genomics. 2014 Jun 18;15(1):488. doi: 10.1186/1471-2164-15-488 (PMC4082626; doi:10.1186/1471-2164-15-488)
Supplement: Supplementary file 11 — Additional file 11: Table S8: miRNA target genes enriched in KEGG pathways. ‘Counts’ means the number of target genes that mapped to the corresponding pathway. (DOCX 38 KB) [file 12864_2014_6194_MOESM11_ESM.docx]

Table S8. The miRNA target genes enriched KEGG pathway. ‘Counts’ means the numbers of target genes that mapped to the corresponding pathway.

| KEGG_Pathway_Name | Pathway_ID | Class | Counts | Percentage | miRNA | Enrichment_Score |
| --- | --- | --- | --- | --- | --- | --- |
| Regulation of actin cytoskeleton | mmu04810 | Cell Motility | 12 | 2.469136 | miR-363-3p | 4.64 |
| Focal adhesion | mmu04510 | Cell Communication | 11 | 2.263374 | miR-363-3p | 4.25 |
| MAPK signaling pathway | mmu04010 | Signal Transduction | 9 | 1.851852 | miR-363-3p | 0.68 |
| Small cell lung cancer | mmu05222 | Cancers | 8 | 1.646091 | miR-363-3p | 7.51 |
| Phosphatidylinositol signaling system | mmu04070 | Signal Transduction | 8 | 1.646091 | miR-363-3p | 10.29 |
| Ubiquitin mediated proteolysis | mmu04120 | Folding, Sorting and Degradation | 8 | 1.646091 | miR-363-3p | 3.62 |
| Insulin signaling pathway | mmu04910 | Endocrine System | 7 | 1.440329 | miR-363-3p | 2.01 |
| ECM-receptor interaction | mmu04512 | Signaling Molecules and Interaction | 7 | 1.440329 | miR-363-3p | 5.56 |
| Axon guidance | mmu04360 | Development | 7 | 1.440329 | miR-363-3p | 2.43 |
| Glioma | mmu05214 | Cancers | 6 | 1.234568 | miR-363-3p | 5.91 |
| Melanoma | mmu05218 | Cancers | 6 | 1.234568 | miR-363-3p | 4.86 |
| TGF-beta signaling pathway | mmu04350 | Signal Transduction | 6 | 1.234568 | miR-363-3p | 3.22 |
| GnRH signaling pathway | mmu04912 | Endocrine System | 6 | 1.234568 | miR-363-3p | 2.76 |
| mTOR signaling pathway | mmu04150 | Signal Transduction | 6 | 1.234568 | miR-363-3p | 7.68 |
| Adherens junction | mmu04520 | Cell Communication | 6 | 1.234568 | miR-363-3p | 4.53 |
| Long-term potentiation | mmu04720 | Nervous System | 6 | 1.234568 | miR-363-3p | 5.62 |
| Pancreatic cancer | mmu05212 | Cancers | 5 | 1.028807 | miR-363-3p | 2.75 |
| Prostate cancer | mmu05215 | Cancers | 5 | 1.028807 | miR-363-3p | 1.81 |
| Renal cell carcinoma | mmu05211 | Cancers | 5 | 1.028807 | miR-363-3p | 2.98 |
| Inositol phosphate metabolism | mmu00562 | Carbohydrate Metabolism | 5 | 1.028807 | miR-363-3p | 5.26 |
| Chronic myeloid leukemia | mmu05220 | Cancers | 4 | 0.823045 | miR-363-3p | 1.21 |
| Non-small cell lung cancer | mmu05223 | Cancers | 4 | 0.823045 | miR-363-3p | 2.6 |
| T cell receptor signaling pathway | mmu04660 | Immune System | 4 | 0.823045 | miR-363-3p | 0.63 |
| Wnt signaling pathway | mmu04310 | Signal Transduction | 4 | 0.823045 | miR-363-3p | 0.16 |
| Neuroactive ligand-receptor interaction | mmu04080 | Signaling Molecules and Interaction | 4 | 0.823045 | miR-363-3p | 1.08 |
| Melanogenesis | mmu04916 | Endocrine System | 4 | 0.823045 | miR-363-3p | 0.53 |
| Cell cycle | mmu04110 | Cell Growth and Death | 4 | 0.823045 | miR-363-3p | 0.3 |
| Colorectal cancer | mmu05210 | Cancers | 3 | 0.617284 | miR-363-3p | 0.12 |
| Acute myeloid leukemia | mmu05221 | Cancers | 3 | 0.617284 | miR-363-3p | 0.79 |
| Endometrial cancer | mmu05213 | Cancers | 3 | 0.617284 | miR-363-3p | 1.04 |
| ErbB signaling pathway | mmu04012 | Signal Transduction | 3 | 0.617284 | miR-363-3p | 0.1 |
| p53 signaling pathway | mmu04115 | Cell Growth and Death | 3 | 0.617284 | miR-363-3p | 0.58 |
| Adipocytokine signaling pathway | mmu04920 | Endocrine System | 3 | 0.617284 | miR-363-3p | 0.41 |
| Dorso-ventral axis formation | mmu04320 | Development | 3 | 0.617284 | miR-363-3p | 3.49 |
| Jak-STAT signaling pathway | mmu04630 | Signal Transduction | 2 | 0.411523 | miR-363-3p | 0.82 |
| Gap junction | mmu04540 | Cell Communication | 2 | 0.411523 | miR-363-3p | 0.02 |
| MAPK signaling pathway | mmu04010 | Signal Transduction | 24 | 3.283174 | miR-302b-5p | 14 |
| Wnt signaling pathway | mmu04310 | Signal Transduction | 17 | 2.325581 | miR-302b-5p | 14.93 |
| Regulation of actin cytoskeleton | mmu04810 | Cell Motility | 15 | 2.051984 | miR-302b-5p | 4.73 |
| Colorectal cancer | mmu05210 | Cancers | 11 | 1.504788 | miR-302b-5p | 11.12 |
| Neuroactive ligand-receptor interaction | mmu04080 | Signaling Molecules and Interaction | 11 | 1.504788 | miR-302b-5p | 0.36 |
| Adherens junction | mmu04520 | Cell Communication | 11 | 1.504788 | miR-302b-5p | 13.83 |
| Melanogenesis | mmu04916 | Endocrine System | 11 | 1.504788 | miR-302b-5p | 8.74 |
| Gap junction | mmu04540 | Cell Communication | 11 | 1.504788 | miR-302b-5p | 9.93 |
| Prostate cancer | mmu05215 | Cancers | 10 | 1.367989 | miR-302b-5p | 7.97 |
| TGF-beta signaling pathway | mmu04350 | Signal Transduction | 10 | 1.367989 | miR-302b-5p | 7.97 |
| Focal adhesion | mmu04510 | Cell Communication | 9 | 1.23119 | miR-302b-5p | 0.6 |
| Ubiquitin mediated proteolysis | mmu04120 | Folding, Sorting and Degradation | 9 | 1.23119 | miR-302b-5p | 2.63 |
| Pancreatic cancer | mmu05212 | Cancers | 8 | 1.094391 | miR-302b-5p | 6.1 |
| Chronic myeloid leukemia | mmu05220 | Cancers | 8 | 1.094391 | miR-302b-5p | 5.68 |
| GnRH signaling pathway | mmu04912 | Endocrine System | 8 | 1.094391 | miR-302b-5p | 3.59 |
| Long-term potentiation | mmu04720 | Nervous System | 8 | 1.094391 | miR-302b-5p | 7.44 |
| Melanoma | mmu05218 | Cancers | 7 | 0.957592 | miR-302b-5p | 4.41 |
| Insulin signaling pathway | mmu04910 | Endocrine System | 7 | 0.957592 | miR-302b-5p | 0.71 |
| Jak-STAT signaling pathway | mmu04630 | Signal Transduction | 7 | 0.957592 | miR-302b-5p | 0.46 |
| Endometrial cancer | mmu05213 | Cancers | 6 | 0.820793 | miR-302b-5p | 4.83 |
| ErbB signaling pathway | mmu04012 | Signal Transduction | 6 | 0.820793 | miR-302b-5p | 1.78 |
| Hedgehog signaling pathway | mmu04340 | Signal Transduction | 6 | 0.820793 | miR-302b-5p | 4.68 |
| Axon guidance | mmu04360 | Development | 6 | 0.820793 | miR-302b-5p | 0.38 |
| Cell cycle | mmu04110 | Cell Growth and Death | 6 | 0.820793 | miR-302b-5p | 0.83 |
| Renal cell carcinoma | mmu05211 | Cancers | 5 | 0.683995 | miR-302b-5p | 1.57 |
| Basal cell carcinoma | mmu05217 | Cancers | 5 | 0.683995 | miR-302b-5p | 2.59 |
| Thyroid cancer | mmu05216 | Cancers | 5 | 0.683995 | miR-302b-5p | 7.63 |
| Glioma | mmu05214 | Cancers | 4 | 0.547196 | miR-302b-5p | 0.89 |
| Small cell lung cancer | mmu05222 | Cancers | 4 | 0.547196 | miR-302b-5p | 0.16 |
| Acute myeloid leukemia | mmu05221 | Cancers | 4 | 0.547196 | miR-302b-5p | 1.08 |
| Non-small cell lung cancer | mmu05223 | Cancers | 3 | 0.410397 | miR-302b-5p | 0.4 |
| Bladder cancer | mmu05219 | Cancers | 3 | 0.410397 | miR-302b-5p | 0.89 |
| T cell receptor signaling pathway | mmu04660 | Immune System | 3 | 0.410397 | miR-302b-5p | 0.03 |
| Phosphatidylinositol signaling system | mmu04070 | Signal Transduction | 3 | 0.410397 | miR-302b-5p | 0.04 |
| Adipocytokine signaling pathway | mmu04920 | Endocrine System | 3 | 0.410397 | miR-302b-5p | 0.04 |
| Circadian rhythm | mmu04710 | Environmental Adaptation | 3 | 0.410397 | miR-302b-5p | 5.84 |
| Inositol phosphate metabolism | mmu00562 | Carbohydrate Metabolism | 2 | 0.273598 | miR-302b-5p | 0.2 |
| Regulation of actin cytoskeleton | mmu04810 | Cell Motility | 18 | 3.474903 | miR-302a-3p | 12.74 |
| Focal adhesion | mmu04510 | Cell Communication | 17 | 3.281853 | miR-302a-3p | 12.67 |
| MAPK signaling pathway | mmu04010 | Signal Transduction | 16 | 3.088803 | miR-302a-3p | 5.85 |
| Chronic myeloid leukemia | mmu05220 | Cancers | 13 | 2.509653 | miR-302a-3p | 26.21 |
| Pancreatic cancer | mmu05212 | Cancers | 12 | 2.316602 | miR-302a-3p | 22.87 |
| Wnt signaling pathway | mmu04310 | Signal Transduction | 12 | 2.316602 | miR-302a-3p | 7.93 |
| Jak-STAT signaling pathway | mmu04630 | Signal Transduction | 11 | 2.123552 | miR-302a-3p | 5.74 |
| Cell cycle | mmu04110 | Cell Growth and Death | 11 | 2.123552 | miR-302a-3p | 10.07 |
| Colorectal cancer | mmu05210 | Cancers | 10 | 1.930502 | miR-302a-3p | 11.69 |
| Renal cell carcinoma | mmu05211 | Cancers | 10 | 1.930502 | miR-302a-3p | 15.59 |
| Small cell lung cancer | mmu05222 | Cancers | 10 | 1.930502 | miR-302a-3p | 11.28 |
| TGF-beta signaling pathway | mmu04350 | Signal Transduction | 10 | 1.930502 | miR-302a-3p | 10.89 |
| Prostate cancer | mmu05215 | Cancers | 9 | 1.737452 | miR-302a-3p | 8.25 |
| ErbB signaling pathway | mmu04012 | Signal Transduction | 9 | 1.737452 | miR-302a-3p | 8.73 |
| Axon guidance | mmu04360 | Development | 9 | 1.737452 | miR-302a-3p | 4.27 |
| Glioma | mmu05214 | Cancers | 8 | 1.544402 | miR-302a-3p | 10.45 |
| Insulin signaling pathway | mmu04910 | Endocrine System | 8 | 1.544402 | miR-302a-3p | 2.43 |
| Ubiquitin mediated proteolysis | mmu04120 | Folding, Sorting and Degradation | 8 | 1.544402 | miR-302a-3p | 2.82 |
| Non-small cell lung cancer | mmu05223 | Cancers | 7 | 1.351351 | miR-302a-3p | 9.64 |
| Melanoma | mmu05218 | Cancers | 7 | 1.351351 | miR-302a-3p | 6.13 |
| mTOR signaling pathway | mmu04150 | Signal Transduction | 7 | 1.351351 | miR-302a-3p | 9.64 |
| Adherens junction | mmu04520 | Cell Communication | 7 | 1.351351 | miR-302a-3p | 5.72 |
| Melanogenesis | mmu04916 | Endocrine System | 7 | 1.351351 | miR-302a-3p | 3.38 |
| ECM-receptor interaction | mmu04512 | Signaling Molecules and Interaction | 6 | 1.158301 | miR-302a-3p | 2.9 |
| Acute myeloid leukemia | mmu05221 | Cancers | 5 | 0.965251 | miR-302a-3p | 3.34 |
| T cell receptor signaling pathway | mmu04660 | Immune System | 5 | 0.965251 | miR-302a-3p | 1.17 |
| p53 signaling pathway | mmu04115 | Cell Growth and Death | 5 | 0.965251 | miR-302a-3p | 2.79 |
| Adipocytokine signaling pathway | mmu04920 | Endocrine System | 5 | 0.965251 | miR-302a-3p | 2.34 |
| Neuroactive ligand-receptor interaction | mmu04080 | Signaling Molecules and Interaction | 5 | 0.965251 | miR-302a-3p | 0.91 |
| Bladder cancer | mmu05219 | Cancers | 4 | 0.772201 | miR-302a-3p | 3.22 |
| Endometrial cancer | mmu05213 | Cancers | 4 | 0.772201 | miR-302a-3p | 2.12 |
| Thyroid cancer | mmu05216 | Cancers | 4 | 0.772201 | miR-302a-3p | 5.66 |
| Basal cell carcinoma | mmu05217 | Cancers | 3 | 0.579151 | miR-302a-3p | 0.66 |
| Phosphatidylinositol signaling system | mmu04070 | Signal Transduction | 3 | 0.579151 | miR-302a-3p | 0.22 |
| GnRH signaling pathway | mmu04912 | Endocrine System | 3 | 0.579151 | miR-302a-3p | 0.2 |
| Gap junction | mmu04540 | Cell Communication | 3 | 0.579151 | miR-302a-3p | 0.12 |
| Hedgehog signaling pathway | mmu04340 | Signal Transduction | 2 | 0.3861 | miR-302a-3p | 0.07 |
| Wnt signaling pathway | mmu04310 | Signal Transduction | 11 | 2.676399 | miR-291a-5p | 9.33 |
| Colorectal cancer | mmu05210 | Cancers | 9 | 2.189781 | miR-291a-5p | 12.53 |
| MAPK signaling pathway | mmu04010 | Signal Transduction | 9 | 2.189781 | miR-291a-5p | 1.18 |
| ErbB signaling pathway | mmu04012 | Signal Transduction | 8 | 1.946472 | miR-291a-5p | 9.15 |
| Focal adhesion | mmu04510 | Cell Communication | 8 | 1.946472 | miR-291a-5p | 1.84 |
| Regulation of actin cytoskeleton | mmu04810 | Cell Motility | 8 | 1.946472 | miR-291a-5p | 1.41 |
| Ubiquitin mediated proteolysis | mmu04120 | Folding, Sorting and Degradation | 7 | 1.703163 | miR-291a-5p | 3.05 |
| Basal cell carcinoma | mmu05217 | Cancers | 6 | 1.459854 | miR-291a-5p | 8.35 |
| TGF-beta signaling pathway | mmu04350 | Signal Transduction | 6 | 1.459854 | miR-291a-5p | 3.99 |
| Neuroactive ligand-receptor interaction | mmu04080 | Signaling Molecules and Interaction | 6 | 1.459854 | miR-291a-5p | 0.12 |
| Endometrial cancer | mmu05213 | Cancers | 5 | 1.216545 | miR-291a-5p | 5.71 |
| T cell receptor signaling pathway | mmu04660 | Immune System | 5 | 1.216545 | miR-291a-5p | 2.07 |
| Adherens junction | mmu04520 | Cell Communication | 5 | 1.216545 | miR-291a-5p | 3.32 |
| Axon guidance | mmu04360 | Development | 5 | 1.216545 | miR-291a-5p | 0.92 |
| Melanogenesis | mmu04916 | Endocrine System | 5 | 1.216545 | miR-291a-5p | 1.88 |
| Cell cycle | mmu04110 | Cell Growth and Death | 5 | 1.216545 | miR-291a-5p | 1.45 |
| Chronic myeloid leukemia | mmu05220 | Cancers | 4 | 0.973236 | miR-291a-5p | 1.59 |
| Renal cell carcinoma | mmu05211 | Cancers | 4 | 0.973236 | miR-291a-5p | 1.9 |
| Small cell lung cancer | mmu05222 | Cancers | 4 | 0.973236 | miR-291a-5p | 1.15 |
| Insulin signaling pathway | mmu04910 | Endocrine System | 4 | 0.973236 | miR-291a-5p | 0.09 |
| GnRH signaling pathway | mmu04912 | Endocrine System | 4 | 0.973236 | miR-291a-5p | 0.86 |
| ECM-receptor interaction | mmu04512 | Signaling Molecules and Interaction | 4 | 0.973236 | miR-291a-5p | 1.26 |
| Glioma | mmu05214 | Cancers | 3 | 0.729927 | miR-291a-5p | 0.89 |
| Pancreatic cancer | mmu05212 | Cancers | 3 | 0.729927 | miR-291a-5p | 0.58 |
| Prostate cancer | mmu05215 | Cancers | 3 | 0.729927 | miR-291a-5p | 0.22 |
| Jak-STAT signaling pathway | mmu04630 | Signal Transduction | 3 | 0.729927 | miR-291a-5p | 0.02 |
| Phosphatidylinositol signaling system | mmu04070 | Signal Transduction | 3 | 0.729927 | miR-291a-5p | 0.63 |
| Long-term potentiation | mmu04720 | Nervous System | 3 | 0.729927 | miR-291a-5p | 0.82 |
| Non-small cell lung cancer | mmu05223 | Cancers | 2 | 0.486618 | miR-291a-5p | 0.18 |
| Melanoma | mmu05218 | Cancers | 2 | 0.486618 | miR-291a-5p | 0.13 |
| Acute myeloid leukemia | mmu05221 | Cancers | 2 | 0.486618 | miR-291a-5p | 0.06 |
| p53 signaling pathway | mmu04115 | Cell Growth and Death | 2 | 0.486618 | miR-291a-5p | 0.03 |
| Circadian rhythm | mmu04710 | Environmental Adaptation | 2 | 0.486618 | miR-291a-5p | 3.43 |
| MAPK signaling pathway | mmu04010 | Signal Transduction | 23 | 2.798054 | miR-290-3p | 6.45 |
| Regulation of actin cytoskeleton | mmu04810 | Cell Motility | 23 | 2.798054 | miR-290-3p | 10.49 |
| Focal adhesion | mmu04510 | Cell Communication | 22 | 2.676399 | miR-290-3p | 11 |
| Axon guidance | mmu04360 | Development | 22 | 2.676399 | miR-290-3p | 23.33 |
| TGF-beta signaling pathway | mmu04350 | Signal Transduction | 18 | 2.189781 | miR-290-3p | 24.36 |
| Pancreatic cancer | mmu05212 | Cancers | 17 | 2.068127 | miR-290-3p | 28.35 |
| Chronic myeloid leukemia | mmu05220 | Cancers | 17 | 2.068127 | miR-290-3p | 26.74 |
| Glioma | mmu05214 | Cancers | 16 | 1.946472 | miR-290-3p | 30.16 |
| Prostate cancer | mmu05215 | Cancers | 15 | 1.824818 | miR-290-3p | 15.2 |
| ErbB signaling pathway | mmu04012 | Signal Transduction | 15 | 1.824818 | miR-290-3p | 16.08 |
| Colorectal cancer | mmu05210 | Cancers | 14 | 1.703163 | miR-290-3p | 13.66 |
| Renal cell carcinoma | mmu05211 | Cancers | 14 | 1.703163 | miR-290-3p | 18.61 |
| Melanoma | mmu05218 | Cancers | 14 | 1.703163 | miR-290-3p | 18.21 |
| Cell cycle | mmu04110 | Cell Growth and Death | 14 | 1.703163 | miR-290-3p | 8.73 |
| Insulin signaling pathway | mmu04910 | Endocrine System | 13 | 1.581509 | miR-290-3p | 4.15 |
| Jak-STAT signaling pathway | mmu04630 | Signal Transduction | 13 | 1.581509 | miR-290-3p | 3.38 |
| Small cell lung cancer | mmu05222 | Cancers | 12 | 1.459854 | miR-290-3p | 8.59 |
| Wnt signaling pathway | mmu04310 | Signal Transduction | 12 | 1.459854 | miR-290-3p | 2.75 |
| mTOR signaling pathway | mmu04150 | Signal Transduction | 12 | 1.459854 | miR-290-3p | 19.17 |
| Ubiquitin mediated proteolysis | mmu04120 | Folding, Sorting and Degradation | 12 | 1.459854 | miR-290-3p | 3.66 |
| Non-small cell lung cancer | mmu05223 | Cancers | 10 | 1.216545 | miR-290-3p | 12.15 |
| T cell receptor signaling pathway | mmu04660 | Immune System | 10 | 1.216545 | miR-290-3p | 4.26 |
| p53 signaling pathway | mmu04115 | Cell Growth and Death | 10 | 1.216545 | miR-290-3p | 8.72 |
| Adherens junction | mmu04520 | Cell Communication | 10 | 1.216545 | miR-290-3p | 6.92 |
| Acute myeloid leukemia | mmu05221 | Cancers | 9 | 1.094891 | miR-290-3p | 7.74 |
| Endometrial cancer | mmu05213 | Cancers | 8 | 0.973236 | miR-290-3p | 6.76 |
| Bladder cancer | mmu05219 | Cancers | 7 | 0.851582 | miR-290-3p | 6.98 |
| Phosphatidylinositol signaling system | mmu04070 | Signal Transduction | 7 | 0.851582 | miR-290-3p | 2.53 |
| Adipocytokine signaling pathway | mmu04920 | Endocrine System | 7 | 0.851582 | miR-290-3p | 2.53 |
| GnRH signaling pathway | mmu04912 | Endocrine System | 6 | 0.729927 | miR-290-3p | 0.42 |
| ECM-receptor interaction | mmu04512 | Signaling Molecules and Interaction | 6 | 0.729927 | miR-290-3p | 0.82 |
| Long-term potentiation | mmu04720 | Nervous System | 5 | 0.608273 | miR-290-3p | 0.9 |
| Melanogenesis | mmu04916 | Endocrine System | 5 | 0.608273 | miR-290-3p | 0.07 |
| Gap junction | mmu04540 | Cell Communication | 5 | 0.608273 | miR-290-3p | 0.06 |
| Neuroactive ligand-receptor interaction | mmu04080 | Signaling Molecules and Interaction | 4 | 0.486618 | miR-290-3p | 3.96 |
| Circadian rhythm | mmu04710 | Environmental Adaptation | 4 | 0.486618 | miR-290-3p | 8.53 |
| Dorso-ventral axis formation | mmu04320 | Development | 4 | 0.486618 | miR-290-3p | 3.23 |
| Basal cell carcinoma | mmu05217 | Cancers | 3 | 0.364964 | miR-290-3p | 0.08 |
| Thyroid cancer | mmu05216 | Cancers | 3 | 0.364964 | miR-290-3p | 1.17 |
| Hedgehog signaling pathway | mmu04340 | Signal Transduction | 3 | 0.364964 | miR-290-3p | 0.02 |
| Inositol phosphate metabolism | mmu00562 | Carbohydrate Metabolism | 3 | 0.364964 | miR-290-3p | 0.07 |
| MAPK signaling pathway | mmu04010 | Signal Transduction | 20 | 3.003003 | miR-20b-3p | 6.27 |
| Axon guidance | mmu04360 | Development | 17 | 2.552553 | miR-20b-3p | 15.71 |
| Focal adhesion | mmu04510 | Cell Communication | 16 | 2.402402 | miR-20b-3p | 5.86 |
| Regulation of actin cytoskeleton | mmu04810 | Cell Motility | 15 | 2.252252 | miR-20b-3p | 3.71 |
| Chronic myeloid leukemia | mmu05220 | Cancers | 14 | 2.102102 | miR-20b-3p | 21.33 |
| TGF-beta signaling pathway | mmu04350 | Signal Transduction | 13 | 1.951952 | miR-20b-3p | 13.87 |
| Glioma | mmu05214 | Cancers | 12 | 1.801802 | miR-20b-3p | 19.16 |
| Pancreatic cancer | mmu05212 | Cancers | 12 | 1.801802 | miR-20b-3p | 15.4 |
| Jak-STAT signaling pathway | mmu04630 | Signal Transduction | 12 | 1.801802 | miR-20b-3p | 4.05 |
| Prostate cancer | mmu05215 | Cancers | 11 | 1.651652 | miR-20b-3p | 8.87 |
| Melanoma | mmu05218 | Cancers | 11 | 1.651652 | miR-20b-3p | 12.88 |
| Small cell lung cancer | mmu05222 | Cancers | 11 | 1.651652 | miR-20b-3p | 9.23 |
| ErbB signaling pathway | mmu04012 | Signal Transduction | 10 | 1.501502 | miR-20b-3p | 7.24 |
| Insulin signaling pathway | mmu04910 | Endocrine System | 10 | 1.501502 | miR-20b-3p | 2.59 |
| Wnt signaling pathway | mmu04310 | Signal Transduction | 10 | 1.501502 | miR-20b-3p | 2.29 |
| Cell cycle | mmu04110 | Cell Growth and Death | 10 | 1.501502 | miR-20b-3p | 4.53 |
| Colorectal cancer | mmu05210 | Cancers | 9 | 1.351351 | miR-20b-3p | 5.48 |
| Non-small cell lung cancer | mmu05223 | Cancers | 9 | 1.351351 | miR-20b-3p | 12.1 |
| Renal cell carcinoma | mmu05211 | Cancers | 9 | 1.351351 | miR-20b-3p | 7.78 |
| Bladder cancer | mmu05219 | Cancers | 8 | 1.201201 | miR-20b-3p | 12.68 |
| p53 signaling pathway | mmu04115 | Cell Growth and Death | 8 | 1.201201 | miR-20b-3p | 6.41 |
| Ubiquitin mediated proteolysis | mmu04120 | Folding, Sorting and Degradation | 8 | 1.201201 | miR-20b-3p | 1.25 |
| T cell receptor signaling pathway | mmu04660 | Immune System | 7 | 1.051051 | miR-20b-3p | 1.94 |
| Adipocytokine signaling pathway | mmu04920 | Endocrine System | 7 | 1.051051 | miR-20b-3p | 3.72 |
| mTOR signaling pathway | mmu04150 | Signal Transduction | 7 | 1.051051 | miR-20b-3p | 6.32 |
| Acute myeloid leukemia | mmu05221 | Cancers | 6 | 0.900901 | miR-20b-3p | 3.39 |
| Adherens junction | mmu04520 | Cell Communication | 6 | 0.900901 | miR-20b-3p | 2.08 |
| Endometrial cancer | mmu05213 | Cancers | 5 | 0.750751 | miR-20b-3p | 2.42 |
| Phosphatidylinositol signaling system | mmu04070 | Signal Transduction | 5 | 0.750751 | miR-20b-3p | 1.19 |
| GnRH signaling pathway | mmu04912 | Endocrine System | 5 | 0.750751 | miR-20b-3p | 0.32 |
| Neuroactive ligand-receptor interaction | mmu04080 | Signaling Molecules and Interaction | 5 | 0.750751 | miR-20b-3p | 2.13 |
| ECM-receptor interaction | mmu04512 | Signaling Molecules and Interaction | 5 | 0.750751 | miR-20b-3p | 0.66 |
| Gap junction | mmu04540 | Cell Communication | 5 | 0.750751 | miR-20b-3p | 0.45 |
| Circadian rhythm | mmu04710 | Environmental Adaptation | 4 | 0.600601 | miR-20b-3p | 10.54 |
| Melanogenesis | mmu04916 | Endocrine System | 4 | 0.600601 | miR-20b-3p | 0.18 |
| Inositol phosphate metabolism | mmu00562 | Carbohydrate Metabolism | 4 | 0.600601 | miR-20b-3p | 1.32 |
| Basal cell carcinoma | mmu05217 | Cancers | 3 | 0.45045 | miR-20b-3p | 0.16 |
| Hedgehog signaling pathway | mmu04340 | Signal Transduction | 3 | 0.45045 | miR-20b-3p | 0.22 |
| Long-term potentiation | mmu04720 | Nervous System | 3 | 0.45045 | miR-20b-3p | 0.04 |
| MAPK signaling pathway | mmu04010 | Signal Transduction | 4 | 3.508772 | miR-18b-5p | 2.42 |
| Ubiquitin mediated proteolysis | mmu04120 | Folding, Sorting and Degradation | 4 | 3.508772 | miR-18b-5p | 6.6 |
| Focal adhesion | mmu04510 | Cell Communication | 3 | 2.631579 | miR-18b-5p | 1.7 |
| Axon guidance | mmu04360 | Development | 3 | 2.631579 | miR-18b-5p | 3.24 |
| Pancreatic cancer | mmu05212 | Cancers | 2 | 1.754386 | miR-18b-5p | 2.33 |
| Renal cell carcinoma | mmu05211 | Cancers | 2 | 1.754386 | miR-18b-5p | 2.46 |
| Acute myeloid leukemia | mmu05221 | Cancers | 2 | 1.754386 | miR-18b-5p | 3.06 |
| T cell receptor signaling pathway | mmu04660 | Immune System | 2 | 1.754386 | miR-18b-5p | 1.63 |
| p53 signaling pathway | mmu04115 | Cell Growth and Death | 2 | 1.754386 | miR-18b-5p | 2.71 |
| Wnt signaling pathway | mmu04310 | Signal Transduction | 2 | 1.754386 | miR-18b-5p | 0.76 |
| GnRH signaling pathway | mmu04912 | Endocrine System | 2 | 1.754386 | miR-18b-5p | 1.58 |
| mTOR signaling pathway | mmu04150 | Signal Transduction | 2 | 1.754386 | miR-18b-5p | 3.49 |
| Neuroactive ligand-receptor interaction | mmu04080 | Signaling Molecules and Interaction | 2 | 1.754386 | miR-18b-5p | 0 |
| Regulation of actin cytoskeleton | mmu04810 | Cell Motility | 2 | 1.754386 | miR-18b-5p | 0.22 |
| Adherens junction | mmu04520 | Cell Communication | 2 | 1.754386 | miR-18b-5p | 2.29 |
| Dorso-ventral axis formation | mmu04320 | Development | 2 | 1.754386 | miR-18b-5p | 7.47 |
| Cell cycle | mmu04110 | Cell Growth and Death | 2 | 1.754386 | miR-18b-5p | 1.28 |
